# Widespread plant specialization in the polyphagous planthopper *Hyalesthes obsoletus* (Cixiidae), a major vector of stolbur phytoplasma: Evidence of cryptic speciation

**DOI:** 10.1371/journal.pone.0196969

**Published:** 2018-05-08

**Authors:** Andrea Kosovac, Jes Johannesen, Oliver Krstić, Milana Mitrović, Tatjana Cvrković, Ivo Toševski, Jelena Jović

**Affiliations:** 1 Department of Plant Pests, Institute for Plant Protection and Environment, Zemun, Serbia; 2 Institute of Organismic and Molecular Evolution, Mainz University, Mainz, Germany; 3 CABI, Delémont, Switzerland; University of Innsbruck, AUSTRIA

## Abstract

The stolbur phytoplasma vector *Hyalesthes obsoletus* is generally considered as a polyphagous species associated with numerous wild and cultivated plants. However, recent research in southeastern Europe, the distribution centre of *H*. *obsoletus* and the area of most stolbur-inflicted crop diseases, points toward specific host-plant associations of the vector, indicating specific vector-based transmission routes. Here, we study the specificity of populations associated with four host-plants using mitochondrial and nuclear genetic markers, and we evaluate the evolution of host-shifts in *H*. *obsoletus*. Host-plant use was confirmed for *Convolvulus arvensis*, *Urtica dioica*, *Vitex agnus-castus* and *Crepis foetida*. Mitochondrial genetic analysis showed sympatric occurrence of three phylogenetic lineages that were ecologically delineated by host-plant preference, but were morphologically inseparable. Nuclear data supported the existence of three genetic groups (Evanno’s ΔK(3) = 803.72) with average genetic membership probabilities > 90%. While populations associated with *C*. *arvensis* and *U*. *dioica* form a homogenous group, populations affiliated with *V*. *agnus-castus* and *C*. *foetida* constitute two independent plant-associated lineages. The geographical signal permeating the surveyed populations indicated complex diversification processes associated with host-plant selection and likely derived from post-glacial refugia in the eastern Mediterranean. This study provides evidence for cryptic species diversification within *H*. *obsoletus sensu lato*: i) consistent mitochondrial differentiation (1.1–1.5%) among host-associated populations in syntopy and in geographically distant areas, ii) nuclear genetic variance supporting mitochondrial data, and iii) average mitochondrial genetic distances among host-associated meta-populations are comparable to the most closely related, morphologically distinguishable species, i.e., *Hyalesthes thracicus* (2.1–3.3%).

## Introduction

Knowledge of changes in insect genetic structure and the possibility of cryptic divergence are particularly important for pest populations because management strategies must be adapted to the ecological diversity of the pest [1–6]. Several planthopper species of the family Cixiidae (Hemiptera: Auchenorrhyncha: Fulgoromorpha) are vectors of plant-pathogenic phytoplasma bacteria whose host-plant choice determines the epidemiological pathways of the transmitted phytoplasma diseases [7–13]. The most promising method for revealing both the vector’s area of distribution and for predicting the disease impact on the targeted crop in the surrounding natural habitat is an assessment of the plants which simultaneously act as preferred host-plants of the vector and as the pathogen’s inoculum source, i.e., a dual host-plant [7, 14, 15]. Conversely, if host-plant preferences change and cause alterations in the vector’s feeding behavior (e.g. mono-, oligo- or polyphagous), such changes may initiate host-plant specialization and drive populations through successive stages of ambiguous, taxonomically indistinguishable but ecologically adapted populations. Such populations known as "host races", "ecological races" or "biotypes" ultimately lead toward true species status [16–18]. Of primary importance for elucidating the distribution, dispersal, impact and epidemiology of vector-transmitted plant diseases is knowledge of the evolutionary relationship between the vector and its host-plant(s). In a polyphagous vector, this is reflected through levels of host-plant association(s) and the extent of genetic segregation, i.e., specialization of the species in question.

Ever since *Hyalesthes obsoletus* Signoret, 1865 (Hemiptera: Cixiidae) rose to prominence as an important pest vector, inducing biomass losses exceeding agroeconomic thresholds, its role in the dissemination of stolbur phytoplasma ('*Candidatus* Phytoplasma solani', 16Sr XII-A subgroup) [19] has been thoroughly investigated [11, 13, 15, 20–25]. The frequent imprecise use of the term "host-plant"—referring to the multitude of wild and cultivated plants on which *H*. *obsoletus* feeds, developments, reproduces or even on which it is accidentally hosted—has led to a general acceptance of *H*. *obsoletus* being highly polyphagous (general overview given in S1 Table) but confuses which plants are utilized for larval development. The most common and widespread host plants are *Convolvulus arvensis* (field bindweed) and *Urtica dioica* (stinging nettle), both recognized as dual host-plants of primary importance in stolbur phytoplasma epidemiology [14, 25–27]. Each plant harbors a specific stolbur phytoplasma strain, *tuf*-a (type I) in *U*. *dioica* and *tuf*-b (type II) in *C*. *arvensis*, which lead to two independent plant-based epidemiologies [14]. *H*. *obsoletus* populations affiliated with these plants are well studied in central Europe [14, 25, 28], especially along the northernmost border of the species distribution range [7, 15, 29]. Recent research has also confirmed these associations and its epidemiological importance in southeastern Europe [13, 30].

Following the experimental verification of *H*. *obsoletus* as a vector of stolbur [23], research on its behavior and host-plant associations has been done mainly in central and western Europe. However, knowledge about (larval developmental) host-plants in the eastern Mediterranean, the species distribution center [31] remains scarce and neglected. For example, affiliation with *Vitex agnus-castus* as a common host-plant in the European Mediterranean has been neglected for a full century after being reported by Horváth [32], even after its confirmation as a true host-plant in Israel [33]. One reason for the neglect is founded on research focusing on agricultural weeds which act as vectors’ host-plant and inoculum source, rather than on the already known preferred plants from natural ecosystems [31]. Recent experimental verification of *H*. *obsoletus* associated with *V*. *agnus-castus* being a vector in the Montenegrin littoral, and the elucidation of stolbur epidemiological routes commencing from different dual host-plants, has encouraged further research to understand the genetic relationships among diverse host-associated populations [13, 34].

Sympatric co-occurrence of populations feeding on (a combination of) the three aforementioned host-plants is often observed, even in strict syntopy [7, 13, 30]. Unlike sympatry in the Montenegrin coastal zone where triple associations are recorded [13], Sharon et al. [33] documented a predominant use of *V*. *agnus-castus* in the eastern Mediterranean where adult presence on co-occurring *U*. *dioica* and *C*. *arvensis* was either lacking or very rare, respectively. Orenstein et al. [35] explained the absence as a phenological mismatch in relation to the insect’s life cycle. A peculiarity that further contributes to the difficulty of understanding *H*. *obsoletus*' host-plant associations across a wide distribution is the fact that it represents only one of the seven species constituting the *Hyalesthes obsoletus* species group: identical outer morphology, similarities of male genital structures and sympatric occurrences as well as the circum-Mediterranean distribution range of *H*. *obsoletus* that overlaps with *H*. *lacotei* (Dlabola, 1970) in south France, *H*. *thracicus* Hoch, 1986 in north Greece and Turkey, *H*. *yozgaticus* Hoch, 1986 in central Turkey (Anatolia), *H*. *hani* Hoch, 1986 in Lebanon, *H*. *verticillatus* Dlabola, 1994 in Israel and Syria, and *H*. *flavovarius* Kusnezov, 1935 in Uzbekistan [31, 36], has made research and exact delimitation of *H*. *obsoletus* and its developmental host plants difficult.

Studies of the genetic structure of plant-associated populations of *H*. *obsoletus* were initiated after severe outbreaks of stolbur-mediated *Bois Noir* disease of grapevine occurred in Germany, Austria and Switzerland. These outbreaks were linked to the recent northward colonization of new habitats by populations affiliated with *U*. *dioica* and the *in situ* formation of *U*. *dioica* and *C*. *arvensis* associated host-races among the peripheral populations on the species’ northernmost distribution range [7, 15, 25, 28, 37]. A study on the causes of *H*. *obsoletus* population expansion and genetic structure has identified an Israeli population comprised of individuals collected on *Vitex* sp. and *Olea europaea* (used as an outgroup for genetic comparison) as seemingly different from the central and west European populations associated with the traditional hosts *C*. *arvensis* and *U*. *dioica* [7, 15]. In addition, a recent study on the distribution, host association and stolbur phytoplasma vectoring ability of *H*. *obsoletus* populations in Serbia lead to the discovery of a new host association with *Crepis foetida*, stinking hawk’s-beard [38]. The first observation of *H*. *obsoletus* aggregation on *C*. *foetida* was made in 2006 on fallow meadows and recently abandoned arable land in east Serbia, near the Bulgarian border (Toševski I., unpublished data). The association was confirmed in the following years over a wider geographic range and preliminary data showed genetic differentiation of *C*. *foetida* populations relative to the *C*. *arvensis*- and *U*. *dioica*-associated populations [38]. Subsequent preliminary insights into genetic divergence relative to Mediterranean *V*. *agnus-castus*–affiliated populations raised questions regarding the cryptic differentiation potential of *H*. *obsoletus* [34].

On the Balkan Peninsula, the first reliable data regarding *H*. *obsoletus* plant associations [22] designated *C*. *arvensis* both as a suitable adult host-plant and as the source of the vectored pathogen (at that time known as the *stolbur virus*). During the following half century, according to faunistic records and summarized data from museum collections for this region, occurrence of *H*. *obsoletus* was indicated primarily on *Vitex* sp., less frequently *Quercus* sp. and only sporadically *Urtica* sp. and *Convolvulus* sp. [31]. The epidemiological role of the planthopper on the Balkan Peninsula has not been studied. However, an increasing pest potential channelled by global increases in temperature promoting population expansion and host-plant adaptation, has highlighted the importance of understanding the true relations between *H*. *obsoletus* and its developmental host-plants in southeastern Europe, the surmised origin of the recent colonization of western Europe [7, 15]. The ecological traits underlying the differentiation of *H*. *obsoletus* populations affiliated with *C*. *arvensis* and *U*. *dioica* at the northwestern edge of the range in western Europe, specific stolbur strains, different adult flight periods and the existence of two plant-based host races [7, 14, 39], represent natural selection processes driven by host-plant choice. This ostensibly polyphagous inhabitant of xerothermic grasslands, disturbed patches and any other available habitat with a preferred host-plant and an adequate soil for nymphal development connects natural and agricultural ecosystems by guiding semi-delineated stolbur transmission routes, which can be deeply affected by the population’s allegiance toward preferred dual host-plant [40].

The present study evaluates genetic differentiation and demographic history in four plant-associated populations of *H*. *obsoletus* from southeastern Europe with the aim of delineating population (host-plant) specialization and possible cryptic speciation. Using previously published genetic data analyzing the same mtDNA and microsatellite loci as the present study [7, 11, 15], we infer phylogenetic ancestry, population expansions and genetic uniqueness of these populations. We assess the evolution of host-race formation and specialization by separating plant- vs. geographic-based population structure. The insights about genetic distinctness can later be corroborated with other species traits such as acoustic signals, behavioral habits and/or subtle morphological structures and lead to proper species delimitation using an integrative approach [17, 41–44]. By testing the hypothesis that plant-affiliated populations in the species distribution center (*sensu* [31]) are highly specialized, we consider the potential for host race formation at its distribution edge [7].

## Materials and methods

### Sampling localities and sampling scheme

**Fig 1:**
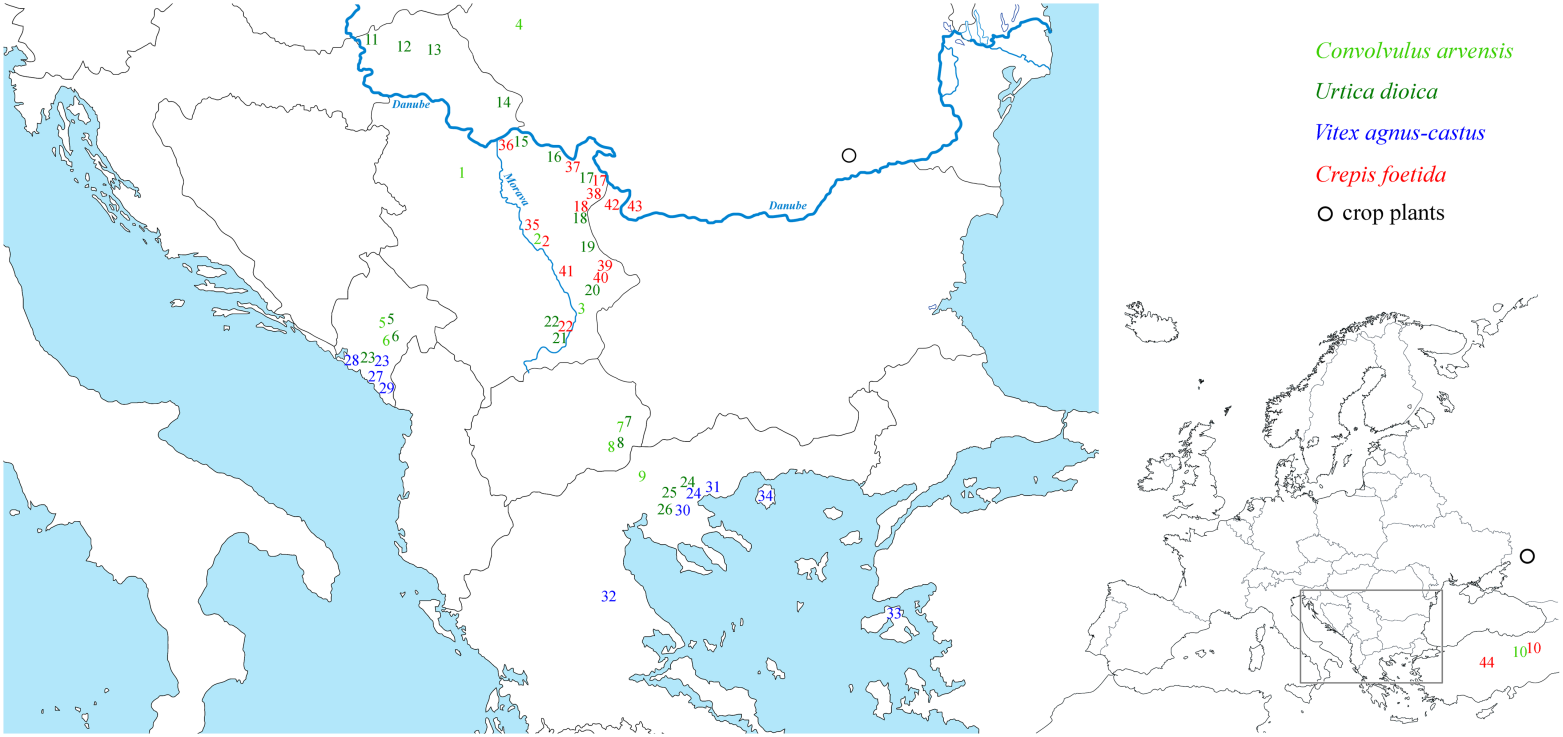
Sampling localities of *Hyalesthes obsoletus* populations and associated host-plants. Numbers refer to localities listed in Table 1. The number’s color refers to the *H*. *obsoletus* population host-plant association as given on the map. Syntopic localities are designated with the same number in two host-plant corresponding colors. The sampling localities of two previously reported *H*. *obsoletus* populations collected on crop plants in Romania and Russia (Radovanu and Mayak) [7] are designated with black outlined circles. Reprinted from d-maps http://d-maps.com/carte.php?num_car=2068&lang=en and http://d-maps.com/carte.php?num_car=2232&lang=en under a CC BY license, with permission from Daniel Dalet, original copyright 2007–2018.

Surveys to collect *Hyalesthes obsoletus* populations associated with four target plants previously identified as preferred plants for this planthopper in southeastern Europe and eastern Mediterranean [13, 22, 31, 33, 38] were performed in 2011–2014 (Fig 1, Table 1). No specific permissions were required, as the study did not involve endangered or protected species. Collections were made from the end of June to the beginning of October, and were based on previous information on adult flight periods in populations associated with each of the four focal host-plants: *Convolvulus arvensis* (*Ca*), *Urtica dioica* (*Ud*), *Vitex agnus-castus* (*Vac*) and *Crepis foetida* (*Cf*) [31, 33, 38, 45]. To evaluate the planthopper’s natural occurrence (regardless of the crop species), we surveyed natural localities such as xerothermic meadows, fallow land, ruderal sites, degraded habitats along roads or rocky substrate of the Mediterranean littoral. At each location, insects were sampled with sweep nets and mouth aspirators from patches of a single host-plant, placed in 2 ml plastic tubes (Sarstedt) filled with 96% ethanol, transported in a portable filled cooler at 10°C to the laboratory and stored at 4°C. All field-collected individuals were examined under a stereomicroscope (Leica MZ7.5) and assigned to *H*. *obsoletus* by white collar and male genital morphology according to the taxonomic key provided by Hoch and Remane [31]. In addition to the sampling in the present study, we included previously reported *H*. *obsoletus* populations collected from crop plants in Romania and Russia (localities Radovanu and Mayak, designated with black outlined circles on Fig 1; [7]) that were not previously tested for mitochondrial diversity.

**Table 1:** Sampled locality data and summarized per population genetic diversity of *Hyalesthes obsoletus* sorted by corresponding host-plant and country of origin.

Host-plant association	Country	Locality no. & name	GPS coordinates	Population no.	nDNA (microsatellite) analyzes				mtDNA analyzes	
					*N*	*A*_*R*_	*H*_*E*_	*F*_*IS*_	haplotype	frequency
*Convolvulus arvensis*	Serbia	1. Topola	N44 13.532 E20 40.224	Pop1	20	4.774	0.791	0.054	EC*	6
		2. Aleksinac	N43 36.010 E21 40.592	Pop2	6	4.626	0.771	-0.015	EC*	6
		3. Predejane	N42 49.992 E22 07.912	Pop3	9	4.956	0.837	0.164	EC*	6
	Romania	4. Petrevo selo	N45 49.528 E21 31.548	Pop4	9	4.763	0.816	0.151	EC*	6
	Montenegro	5. Martinići	N42 32.245 E19 10.763	Pop5	17	4.412	0.777	0.264	AB*	6
		6. Podgorica	N42 26.919 E19 12.509	Pop6	20	4.192	0.748	0.143	AB*	6
	Macedonia	7. Hamzali	N41 29.860 E22 44.996	Pop7	12	4.549	0.787	0.169	EC*	6
		8. Strumica	N41 26.505 E22 39.922	Pop8	11	4.282	0.744	0.146	EC*	4
									πC	1
									ψC	1
	Greece	9. Kilkis	N40 54.984 E22 49.101	Pop9	6	4.550	0.721	0.216	AB*	1
									XB	1
									WL	2
									WB	2
	Turkey	10. Erzincan	N39 57.253 E38 38.063	Pop10	3	-	-	-	SK	3
*Urtica dioica*	Serbia	11. Gakovo	N45 55.880 E19 03.280	Pop11	22	4.937	0.820	0.141	EC*	4
									FC*	1
									RC	1
		12. Bačka Topola	N45 47.518 E19 35.604	Pop12	20	4.934	0.826	0.157	EC*	4
									ωC	2
		13. Bačko Petrovo selo	N45 43.693 E20 06.114	Pop13	20	5.100	0.844	0.155	EC*	6
		14. Vršac	N45 03.874 E21 11.208	Pop14	12	4.590	0.804	0.148	EC*	6
		15. Srednjevo	N44 39.685 E21 30.362	Pop15	15	4.705	0.787	0.087	EC*	6
		16. Boljetin	N44 31.740 E22 02.090	Pop16	12	4.714	0.815	0.212	EC*	6
		17. Negotin	N44 16.604 E22 30.484	Pop17	6	4.644	0.803	-0.003	EC*	6
		18. Zaječar	N43 50.016 E22 17.334	Pop18	20	4.807	0.816	0.072	EC*	4
									ξQ	2
		19. Knjaževac	N43 30.610 E22 18.833	Pop19	15	4.923	0.836	0.226	EC*	6
		20. Grnčar	N43 01.270 E22 21.825	Pop20	17	4.492	0.777	0.130	EC*	6
		21. Vranje	N42 31.725 E21 54.319	Pop21	12	4.380	0.76	0.120	EC*	6
		22. Vranjska banja	N42 34.139 E21 58.367	Pop22	11	4.425	0.753	0.060	EC*	6
	Montenegro	23. Godinje	N42 13.421 E19 06.800	Pop23	6	4.395	0.758	0.166	EC*	6
		5. Martinići	N42 32.245 E19 10.763	Pop24	20	4.306	0.746	0.122	EC*	6
		6. Podgorica	N42 26.919 E19 12.509	Pop25	20	4.362	0.76	0.113	EC*	2
									αC	2
									βC	2
	Macedonia	7. Hamzali	N41 29.860 E22 44.996	Pop26	17	4.091	0.714	0.024	EC*	6
		8. Strumica	N41 26.505 E22 39.922	Pop27	19	4.194	0.735	0.104	EC*	6
	Greece	24. Arethousa	N40 45.767 E23 33.096	Pop28	12	4.007	0.685	-0.060	EC*	6
		25. Filadelfio	N40 45.246 E23 27.846	Pop29	20	3.500	0.622	-0.009	EC*	6
		26. Profitis	N40 39.930 E23 17.331	Pop30	6	3.602	0.643	0.193	EC*	3
									WB	2
									ρC	1
*Vitex agnus-castus*	Montenegro	27. Bar	N42 07.031 E19 04.581	Pop31	18	4.198	0.747	0.038	ZN	5
									γN	1
		23. Godinje	N42 13.421 E19 06.800	Pop32	19	4.590	0.803	0.101	ηN	6
		28. Kamenari	N42 28.591 E18 41.028	Pop33	20	3.667	0.67	0.059	ZN	5
									ZO	1
		29. Ulcinj	N41 56.515 E19 16.052	Pop34	19	4.753	0.817	0.163	ZN	2
									ηN	2
									θN	2
	Greece	30. Apollonia	N40 38.380 E23 29.960	Pop35	10	4.732	0.819	0.168	YM	6
		31. Asprovalta	N40 45.124 E23 44.024	Pop36	19	4.771	0.795	0.176	YM	6
		24. Arethousa	N40 45.767 E23 33.096	Pop37	20	5.154	0.855	0.204	YM	4
									ηM	2
		32. Larisa	N39 38.544 E22 16.981	Pop38	7	4.320	0.775	0.065	YM	6
		33. Lesbos	N39 18.565 E26 8.379	Pop39	5	-	-	-	YM	3
									σM	2
		34. Thasos	N40 35.177 E24 37.525	Pop40	3	-	-	-	YM	3
*Crepis foetida*	Serbia	2. Aleksinac	N43 36.010 E21 40.592	Pop41	10	3.430	0.689	0.388	JH	4
									MH	2
		35. Deligrad	N43 38.546 E21 33.444	Pop42	20	3.552	0.688	0.193	JH	2
									MH	2
									μH	2
		36. Požarevac	N44 39.310 E21 11.983	Pop43	12	3.334	0.638	0.181	JH	4
									MH	2
		37. Porečka reka	N44 24.267 E22 10.350	Pop44	4	-	-	-	JH	1
									MH	1
									UH	1
									VH	1
		17. Negotin	N44 16.604 E22 30.484	Pop45	21	3.323	0.619	0.282	JH	3
									MH	3
		38. Tamnič	N44 04.973 E22 32.048	Pop46	7	3.365	0.613	0.165	JH	4
									MH	2
		18. Zaječar	N43 50.016 E22 17.334	Pop47	6	3.329	0.602	0.363	JH	4
									MH	2
		39. Temska	N43 16.537 E22 31.840	Pop48	12	3.370	0.656	0.200	JH	6
		40. Pirot	N43 12.880 E22 31.585	Pop49	19	3.689	0.687	0.257	JH	6
		41. Jasenovik	N43 22.365 E22 02.441	Pop50	20	3.743	0.715	0.147	JH	6
		22. Vranjska banja	N42 34.139 E21 58.367	Pop51	7	3.067	0.598	0.370	JH	2
									MH	2
									λH	2
	Bulgaria	42. Vidin	N43 57.711 E22 51.258	Pop52	6	2.800	0.563	0.198	JH	4
									MH	2
	Romania	43. Calafat	N43 59.926 E22 58.119	Pop53	10	3.452	0.631	0.305	JH	4
									MH	2
	Turkey	10. Erzincan	N39 57.253 E38 38.063	Pop54	8	4.379	0.768	0.175	JH	6
		44. Kırşehir	N39 26.021 E34 07.795	Pop55	1	-	-	-	JH	1

Sample size (*N*); Allelic richness (*A*_*R*_); Expected heterozygosity (*H*_*E*_); Inbreeding coefficient (*F*_*IS*_).

Symbol "-" refers to genetic indices that are not calculated due to small sample size.

Mitochondrial (mtDNA) haplotypes designation according to Johannesen et al. [15]; First letter in haplotype name corresponds to *COI*-*tRNA(Leu)*-*COII* gene region haplotype designation and second letter to the *16S*-*tRNA(Leu)*-*ND1* gene region haplotype.

MtDNA haplotypes described in previous studies [8, 11, 15, 37] are denoted with asterisk "*".

### DNA extraction

Total DNA was extracted from each individual insect specimen using a non-destructive, partly modified sodium dodecyl sulfate (SDS) extraction method [46, 47]. Briefly, all specimens were punctured between hind legs and mesothorax and incubated overnight at 56°C in extraction buffer (SDS 0.5%, Tris 20 mM, EDTA 10 mM) with proteinase K (Fermentas) at a concentration of 187.5 μg/mL^-1^. After removing the insect, chloroform was added to the homogenate. The mixture was centrifuged at 4°C on 11000 rpm for 10 min. The chloroform step was repeated, after which the upper aqueous supernatant was precipitated with ice-cold isopropanol. This mixture was centrifuged at maximum speed for 15 min. The resulting DNA pellet was washed with 96% ethanol, air dried and re-suspended in 50 μl TE buffer (10 mM Tris, 1 mM EDTA, pH 7.6). To achieve a higher rate of DNA recovery for the dry museum specimen, and for specimens from localities in Turkey, Greece and Serbia with only few individuals, we used the Qiagen Dneasy Blood & Tissue Kit (Hilden, Germany) in accordance with the manufacturer’s instructions. Both extraction methods allowed us to preserve morphological features of specimens for later analyses. After DNA extraction, insect specimens were prepared as voucher dry specimens. They are housed at the Institute for Plant Protection and Environment collection (IPPE, Zemun, Serbia). Extracted DNA was kept at -20°C.

### Mitochondrial genotyping

**Fig 2:**
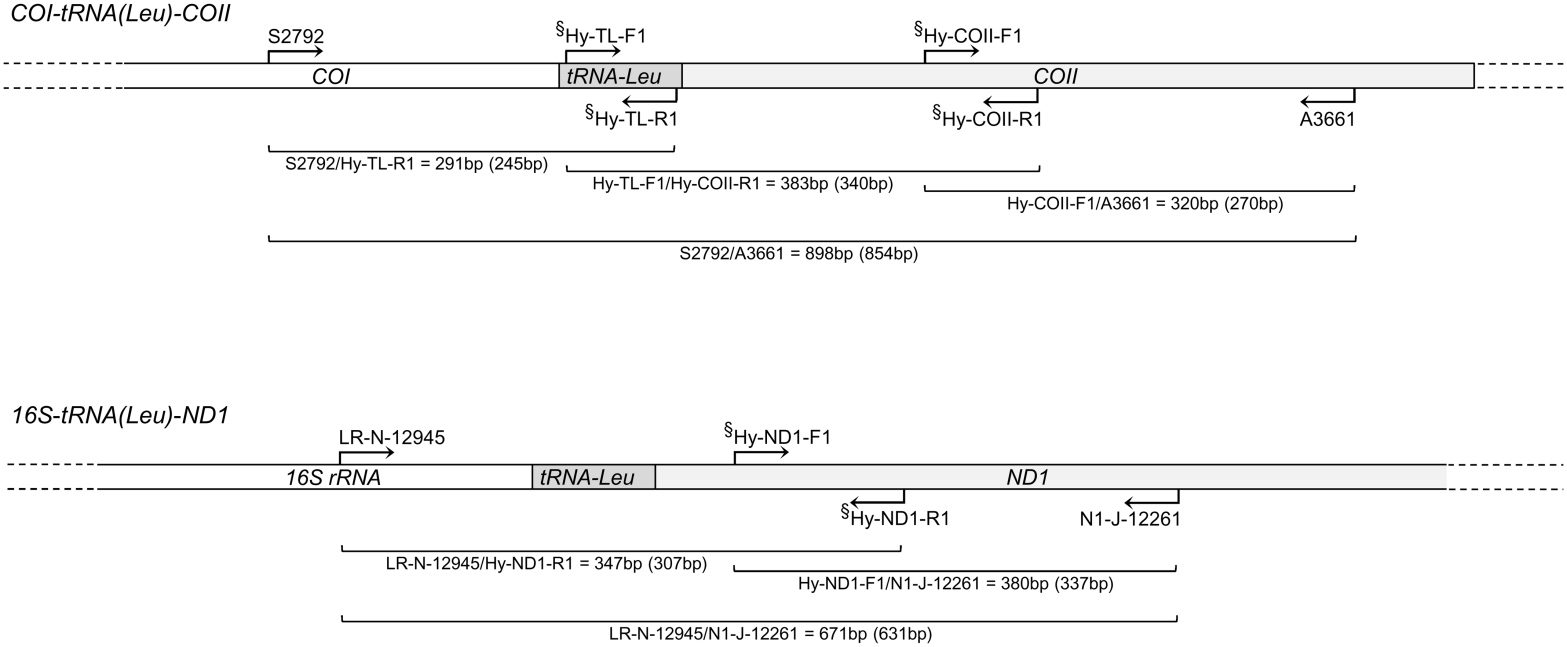
Schematic representation of *COI*-*tRNA(Leu)*-*COII* and *16S*-*tRNA(Leu)*-*ND1* mtDNA gene regions showing the binding site positions of the primers used for amplification of freshly collected *Hyalesthes obsoletus* specimens and dry museum *H*. *thracicus* specimen. The amplicon length (bp) for each primer pair combination is given below the scheme (length excluding primers is given in parentheses). Primers marked with the symbol "§" were designed in this study and used for the amplification of short DNA fragments of the *H*. *thracicus* paratype specimen. Scheme not drawn to scale. Primer sequences are given in S2 Table.

Mitochondrial DNA analyses of *H*. *obsoletus* were based on two concatenated gene regions (markers) previously characterized by Johannesen et al. [15, 37]: (i) cytochrome oxidase subunit I, tRNA for leucine and cytochrome oxidase subunit II region (*COI-tRNA(Leu)-COII*) and (ii) 16S ribosomal RNA, *tRNA-Leu* and reduced nicotinamide adenine dinucleotide (NADH) dehydrogenase subunit I region (*16S-tRNA(Leu)-ND1*). Amplifications were performed using primers S2792 and A3661 for the first gene region [48] and LR-N-12945 and N1-J-12261 for the second region [49] (Fig 2, S2 Table). Polymerase chain reactions (PCR) for both markers were performed in a 20-μL final reaction volume using Kapa Biosystems High Yield Reaction Buffer A (1.5 mM MgCl2, 1×), an additional 3.5 mM of MgCl2 for amplification of *COI-tRNA(Leu)-COII* and 1.5 mM for *16S-tRNA(Leu)-ND1*, 0.5 mM of each dNTP, 0.4 μM of each primer, 1 U of KAPA *Taq* DNA polymerase (Kapa Biosystems, Inc., Woburn, MA, USA) and 1 μL of template DNA. Amplification was conducted in a Mastercycler ep gradient S (Eppendorf, Hamburg, Germany) using the following thermal profile for both markers: 95°C for 2 min, 35 cycles at 95°C for 30 s, 48°C for 1 min, 72°C for 1 min 30 s, and a final extension at 72°C for 10 min. Sequencing was performed on an ABI Prism 3700 automated sequencer (Macrogen Inc., Seoul, South Korea). Sequences were edited using FinchTV v.1.4.0 (http://www.geospiza.com) and aligned with ClustalW [50] within the MEGA v.5.2 software [51]. Whenever possible, six specimens per each *H*. *obsoletus* population were genotyped for mitochondrial markers, (Table 1). The nomenclature of newly identified haplotypes followed the designation system established by Johannesen et al. [15, 37]. Haplotypic nucleotide sequences were submitted to NCBI GenBank under the accession numbers KY368699-724 for *COI-tRNA(Leu)-COII* and KY368692-8 for *16S-tRNA(Leu)-ND1* (S3 Table).

Considering that orthology is of primary importance for phylogenetic studies, especially when cryptic speciation events are possibly involved [52], we performed analyses to confirm the orthologus status (and rule out nuclear pseudogenes of mitochondrial origin, Numts) of the amplified mitochondrial markers. To obtain reliable orthologous mitochondrial sequence of both marker genes, we made serial dilutions of genomic DNA for a subset of samples prior to performing the described PCR amplification protocols. We considered that in dilution of 1:6,250 any amplification reaction would contain less than one nuclear genome [53]. Sequences obtained from diluted samples were compared with the ones obtained using undiluted genomic DNA to confirm their identity and mitochondrial origin.

To achieve a better resolution for the evolutionary relations between and among *H*. *obsoletus* host-plant associated haplotype groups, we supplemented our study with *Hyalesthes thracicus* genetic data, morpho-phylogenetically the closest member of the same species group [31, 54]. We obtained an archival, museum, male paratype specimen collected in 1979 in the Lake Volvi surroundings (Greece), deposited in Prof. H. Hoch’s private collection (S1 Fig). The entomological tile and glue were carefully removed, where after the paratype was prepared for extraction as previously described. Considering that mtDNA in dry insect material is frequently highly degraded and fragmented, the archival specimen was analyzed using a set of four and two degenerate primers designed to amplify short fragments of the *COI-tRNA(Leu)-COII* and *16S-tRNA(Leu)-ND1* gene regions, respectively (Fig 2, S2 Table). Newly designed primers were degenerate due to an unknown sequence at the primer binding site. The primer design was based on a sequence alignment comparison among phylogenetically closely and more distantly related cixiid planthoppers: *Hyalesthes philesakis* Hoch, 1986, *H*. *luteipes* Fieber, 1876, *H*. *ponticorum* Hoch, 1986, *H*. *aylanus* Hoch, 1986, *Reptalus panzeri* (Löw, 1883), *R*. *melanochaetus* (Fieber, 1876), *R*. *cuspidatus* (Fieber, 1876) and *Setapius apiculatus* (Fieber, 1876). Six newly designed primers were combined with standard ones [48, 49], producing overlapping amplicons that allowed the recovery of the entire genetic region analyzed for fresh material (Fig 2; S2 Table). PCR conditions for short fragment amplification were the same as described for the full-length marker genes with the exception of an extension time that was reduced to 45 s. Nucleotide sequences of the *H*. *thracicus COI-tRNA(Leu)-COII* and *16S-tRNA(Leu)-ND1* gene regions were deposited in GenBank under the numbers KY368725 and KY368726, respectively.

### Mitochondrial diversity and population differentiation

Genetic variations within and among host-plant associations were estimated with pairwise *F*-statistics [55] implemented in Arlequin v.3.5.1.2 [56]. Mitochondrial haplotype diversity was calculated for each of the 55 *H*. *obsoletus* populations (number and frequency of haplotypes). Standard molecular diversity indices (*F*_*ST*_ and θ) were calculated for the three metapopulations that were grouped according to the host-plant association and the cluster sharing between the populations associated with *C*. *arvensis* and *U*. *dioica* as host-plants: 1) *C*. *arvensis* + *U*. *dioica*, 177 individuals; 2) *V*. *agnus-castus*, 56 individuals; and 3) *C*. *foetida*, 83 individuals. To estimate the amount of genetic diversity within the selected groups (metapopulations) we calculated the population genetic parameter theta (θ) estimated from the number of alleles (θ_k_), expected homozygosity (θ_H_), segregating sites (θ_S_) and pairwise differences (θ_π_). Theta (θ) represents the distribution of variation within or among populations when samples are considered to represent characteristics of the higher rank group from which they are sampled, in this case, the host-plant association [57, 58].

To study whether genetic diversity of each of the four host-plant associated groups depart from neutrality and to detect genetic signals of the demographic and/or spatial growth we calculated Tajima’s *D* [58] and Fu’s *F*_*S*_ neutrality statistics [59]. Neutrality tests were performed in Arlequin v.3.5.1.2 [56]. Because *U*. *dioica*-associated populations are known to be undergoing demographic expansion in western Europe [8, 37], individuals associated with *C*. *arvensis* and *U*. *dioica* were treated both separately and as a meta-group to deduce the source of a departure from neutrality. Fu’s simulations are based on the infinite sites model of mutation. Negative values of Fu’s *F*_*S*_ parameter indicate an excess in allele numbers as expected from a recent population expansion, while a positive value is evidence of an allele deficiency, as expected from a recent population bottleneck [60]. Tajima’s *D* test measures a difference in the number of pairwise differences and the number of segregating sites scaled to be the same in a neutrally evolving population. A negative Tajima’s *D* parameter signifies an excess of low frequency polymorphisms. A significant negative departure from these tests has been explained mainly as an excess of new mutations as a result of evolutionary forces, such as selective sweeps or population growth (signature of population expansion), while positive departure indicate a balancing selection.

The pairwise fixation index (*F*_*ST*_) values and the Nei’s average number of pairwise differences [61] were calculated using Arlequin, to measure differentiation between all population pairs sorted according to host-plant association (as presented in Table 1). In total, calculations were performed for 51 *H*. *obsoletus* populations harboring at least five individuals. *F*_*ST*_ values range from 0 (including negative values) to 1, with 0 indicating no divergence between the populations, and 1 indicates that two populations are completely separate [55, 62]. Nei’s pairwise differences assume genetic differences that arise from mutations and changes in the frequency of haplotype in a population due to random sampling.

### Phylogenetic analysis and haplotype network construction

Phylogenetic reconstruction and haplotype networks were based on newly identified haplotypes (this study) and all previously published *H*. *obsoletus* haplotypes [8, 11, 15, 37]. To obtain the best resolution of the phylogenetic relationships among the host-plant associated *H*. *obsoletus* populations, *H*. *thracicus* was used for tree rooting in all mtDNA phylogenetic analyses. Concatenated sequences of both mtDNA gene regions (1180 bp in length) were aligned and treated as a single locus. The maximum parsimony and neighbor-joining trees were generated in PAUP* v.4.0b10 [63] using the evolutionary model of nucleotide substitution that best fit the data, determined with jModelTest v.2.1.7 [64] under unconstrained prior distributions. Five hundred bootstrap replicates were performed to assess the branch support of the resulting tree topologies. The suggested substitution model was also used for the Bayesian-based phylogenetic analysis implemented with MrBayes v.3.1.2 [65, 66]. We used the following settings: two simultaneous runs executed for 1,000,000 generations, sampling every 100 generations and with a ‘burn-in’ of 25%. Posterior probabilities were assessed with Tracer v.1.5.0 [67] to ensure that sampling reached stationarity within the burn-in. The obtained trees were visualized in FigTree v.1.4 [68]. Average genetic distances among haplotypes were calculated within and between major phylogenetic clusters using PAUP* under the nucleotide substitution model as determined by jModelTest found for the phylogenetic tree reconstruction (see above). Additionally, genetic distances were calculated separately for haplotypes of the concatenated *COI-COII* genes, which was proven to be the most phylogenetically informative of the analyzed mtDNA markers for species-level identification and differentiation, e.g., [69–71].

Phylogenetic relationships between closely related species and such resulting from population-level processes (e.g., persistence of ancestral haplotypes, multifurcations, recombination and horizontal transfer) are often better visualized in reticulated graphs or networks [72]. MtDNA gene-region genealogies of *H*. *obsoletus* host-associated haplogroups were inferred employing two software packages with different network construction approaches. The software TCS v.1.21 [73] was used to construct a haplotype network based on statistical parsimony [74] with 95% confidence limits, while a median-joining network was calculated using Network v.4.612 (www.fluxus-engineering.com) keeping the parameter ε = 0. This method adds median vectors as uncollected or possibly extinct ancestral genotypes in order to reduce tree length to the minimum spanning trees combined within a single network [75].

### Microsatellite genotyping and diversity

Microsatellites primers and PCR conditions follow Imo et al. [7, 76] and Maniyar et al. [28]. The microsatellite analysis was performed using a GA3130XL Genetic Analyzer (Applied Biosystems) (Mainz University). In total, 702 *H*. *obsoletus* individuals originating from 50 populations (Table 1) were genotyped at seven microsatellite loci previously used to delimit and to characterize population genetic structure and host races of *H*. *obsoletus* [7]. The loci were genotyped using GeneMapper v.4.0 (Applied Biosystems).

The presence of null alleles was evaluated using the EM algorithm (FreeNa) [77, 78]. Null allele frequencies were calculated per locus and population, as well as overall and per host-plant association. Deviations from Hardy-Weinberg (HW) were estimated using Micro-checker [79]. Linkage disequilibrium (LD) was checked with the web-based version of Genepop [80] using the settings 1,000 batches and 10,000 de-memorizations and iterations per batch. The sex-linked locus C147 was omitted in both analyses. Significance levels for multiple comparisons were adjusted by Bonferroni correction [81]. The allele number (A), the allelic richness (*A*_*R*_) and allele frequencies per locus were estimated in FSTAT v.2.9.3 [82] using all seven loci; whereas C147 was excluded in estimates of the population inbreeding coefficient (*F*_*IS*_). The mean expected heterozygosity per population (*H*_*E*_) was calculated in Arlequin v.3.5.1.2 [56]. Differences in the mean *H*_E_ and *A*_*R*_ per group of the host-plant associated populations were tested with *t*-tests using Statistica v.5.1 (StatSoft, Inc. 1997). Because sample sizes varied, we performed a linear regression analyses to test sample the dependency of sample size and genetic diversity measures.

### Microsatellite population structure and differentiation

Analyses of the influence of host-plant affiliation and geographic separation on population structure were estimated using Bayesian clustering (Structure v.2.3.3) [83]. Following the procedure described in Imo et al. [7], we first estimated the highest level of genetic clustering, K, for all individuals based on their multi-locus genotypes. Alongside the 702 individuals collected in this study, we analyzed a single 20-member population associated with *Vitex agnus-castus* from Israel [7]. Based on the results of overall population clustering and knowing that the Bayesian analysis can be influenced by relative genotype frequencies, which may hide signals of a lower-level structure, we estimated association levels, i.e., K, within clusters for additional effects of geographic distance and host-plant. The Structure analysis for each test was repeated 20 times for each K with 50,000 burn-ins followed by 200,000 MCMC (Markov chain Monte Carlo) iterations using the admixture model and correlated allele frequencies. The web-based program Structure harvester [84] was used for visualizing the likelihood plateau of the distribution of LnP(D) and for inferring the most likely number of genetic clusters (ΔK) according to Evanno et al. [85]. Additionally, syntopic localities in which the geographical signal is excluded as a distortive factor were singled out in separate independent analyses. Genetic differentiation among clusters identified with Structure was estimated with the molecular variance analysis (Amova) in Arlequin v.3.5.1.2 [56].

Genetic clustering and identity among *H*. *obsoletus* populations associated with the four host-plants were further inferred with a maximum-likelihood phenogram using the Contml algorithm in Phylip v.3.69 [86]. *Hyalesthes luteipes* individuals sampled on *Ulmus minor* in Serbia were used as an outgroup root. This analysis was based on four loci (F56, F84, H120, G85) that amplified successfully in both species. The phenogram was visualized using FigTree v.1.4 [68]. It should be noted here that outgroups differed between the mtDNA and microsatellite analyses. MtDNA analysis was based on a single museum specimen of the nearest morphological relative *H*. *thracicus*. In contrast, microsatellite analysis (*H*. *luteipes*) relies on allele frequencies for which estimate several individual are required.

## Results

Target host-plants were searched for associated *H*. *obsoletus* populations across the surveyed area (Fig 1) to obtain information on the geographic range of each insect-host association, distribution overlap (sympatry), population structure and genetic differentiation in syntopy. Surveys indicated that *H*. *obsoletus* populations in southeastern Europe are i) high density and very common in association with *Ud*; ii) rare and low density in association with *Ca*; iii) usually very common and in high density in association with *Vac*, however, it is restricted to the coastal distribution of the host-plant; and iv) high in number and very common in association with *Cf* in the eastern parts of the surveyed area but absent in the south (Fig 1). The number of collected individuals per population was always intended to be greater than or equal 6 and preferably 20. However, on some occasions this number was not achieved due to the end of the adult flight period or a low-density population (Pop10, 39, 40, 44 and 55; Table 1). In total, populations were collected in six countries with at least two identified *H*. *obsoletus* host-plant associations: Serbia (*Ca*, *Ud* and *Cf*), Romania (*Ca* and *Cf*), Montenegro (*Ca*, *Ud* and *Vac*), Macedonia (*Ca* and *Ud*), Greece (*Ca*, *Ud* and *Vac*) and Turkey (*Ca* and *Cf*). In addition to these locations, a single locality with *Cf* as a tentative host-plant was surveyed at a Danube locality in Bulgaria (Fig 1, locality 42) for the purpose of assessing colonization route and the distribution range of *Cf*-associated *H*. *obsoletus* populations. Locality 43 in Romania was surveyed to test whether the Danube acts as a natural barrier to the spread of *Cf*-associated *H*. *obsoletus* population into Central Europe (Fig 1). The collections made in Turkey were performed only on the two listed localities, without surveying other potential host-plants or their surroundings.

A total of 718 *H*. *obsoletus* adults were sampled from 55 populations at 44 localities in southeastern Europe and Turkey (Table 1, Fig 1). Eleven sites held syntopic host-plant populations of the planthopper: four *Ca*/*Ud* in Macedonia and Montenegro, two *Ud*/*Vac* in Greece and Montenegro, two *Cf*/*Ca* in Serbia and Turkey, and three *Cf*/*Ud* in Serbia. Ten *H*. *obsoletus* populations were sampled from *C*. *arvensis*, and populations from *U*. *dioica* were sampled at 20 localities. The *V*. *agnus-castus*-associated populations were sampled from four localities along the coastline area of Montenegro, from six localities in Thessaly (Greece), and from the Aegean islands Thasos and Lesbos 10 km from the nearest Greek or Turkish mainlands, respectively. The affiliation of *H*. *obsoletus* populations with *C*. *foetida* was observed mainly in eastern and southeastern Serbia, with a western limit along the river Morava (N = 11). The association with *C*. *foetida* was confirmed along the Danube in Bulgaria and Romania (Fig 1) but it was not observed in Montenegro, Macedonia or Greece. An additional nine adults were collected from *C*. *foetida* at two sites in Turkey, of which one, located in an eastern part of the Anatolian plateau (locality 10), held a syntopic *C*. *arvensis* population.

### Population genetic diversity based on mitochondrial and microsatellite data

**Fig 3:**
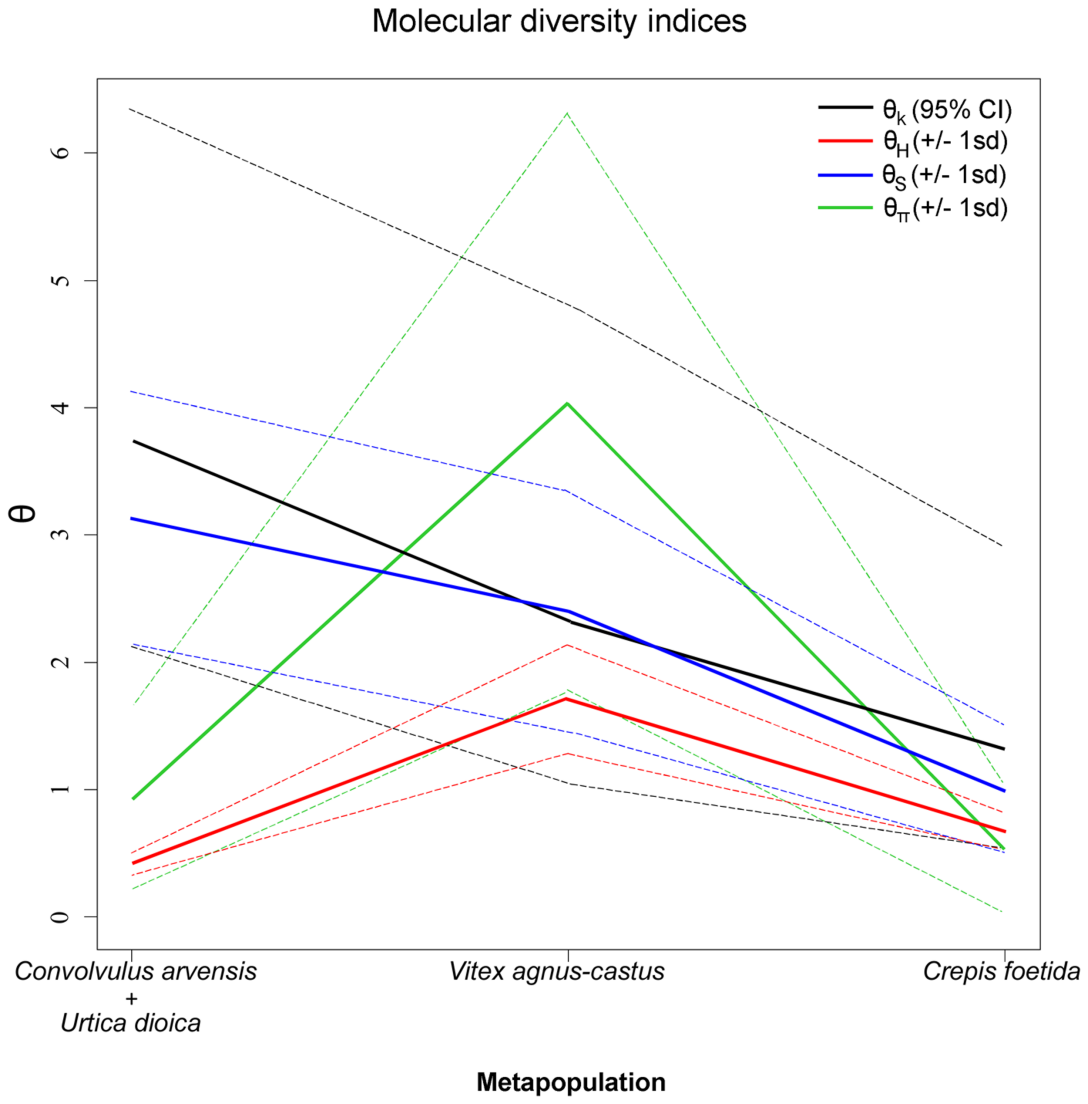
Mitochondrial diversity indices calculated 316 *Hyalesthes obsoletus* genotyped specimens of the three host-associated metapopulations. *Convolvulus arvensis* and *Urtica dioica* (177 specimens), *Vitex agnus-castus* (56 specimens) and *Crepis foetida* (83 specimens). Indices are designated in colors: black = number of alleles (θ_k_), red = expected homozygosity (θ_H_), blue = segregating sites (θ_S_) and green = pairwise differences (θ_π_).

The mitochondrial genetic diversity analysis revealed highly significant differentiation among the three host-plant associated groups (*Ca-Ud*, *Vac* and *Cf* metapopulations), with 88% of genetic variance derived from host-plants (*F*_*ST*_ = 0.88, p < 0.001) and 12% attributed to within-group variations. Diversity estimates within and among host-associated metapopulations (Fig 3) were calculated for 316 individuals from the 55 *H*. *obsoletus* populations collected in our study (Table 1). Twenty-nine haplotypes were identified, three of which were previously found in the *H*. *obsoletus* populations associated with *Ca* and *Ud* from central Europe (AB, EC and FC; [15]), and 26 were newly described haplotypes from southeastern Europe and Turkey. Of the new haplotypes, three were found in association with *Ca*, six with *Ud*, eight with *Vac* and six with *Cf* (S3 Table). Overall, from 177 individuals collected from *Ca-Ud*, 56 from *Vac* and 83 sampled from *Cf*, 15, 8 and 6 haplotypes were recorded in each host-associated metapopulation, respectively.

Haplotypes identified in one of the three delineated metapopulations were never observed in the other two, i.e., haplotypes were host-specific at all localities (Table 1). For the two *H*. *obsoletus* populations collected on crop plants in Romania and Russia (localities Radovanu and Mayak; [7]), the Romanian locality exhibited haplotypes affiliated with both *Ca*-*Ud* (EC, including new haplotypes PC and ΦC) and *Cf* (JH and MH), while the Russian had a single haplotype (QB, S3 Table) derived from the AB haplotype of the *Ca-Ud* metapopulation. These two populations were not included in diversity estimates due to doubtful host-plant origin.

Comparison among the theta (θ) values of molecular diversity in these three host-plant associations revealed the highest mtDNA diversity estimates of number of alleles and segregating sites in the *Ca-Ud H*. *obsoletus* metapopulation (θ_k_ = 3.73 and θ_s_ = 3.13; Fig 3). In case of *Vac*- and *Cf*-associated metapopulations number of alleles θ_k_ was 2.32 and 1.31, and segregating sites θ_s_ was 2.39 and 1.00, respectively. Higher diversity estimates in the *Ca-Ud* metapopulation correlated with departures from the population equilibrium, indicating a recent population expansion, as suggested by the significantly negative values of Fu’s and Tajima’s neutrality indices (*F*_*S*_ = -9.59, p < 0.001; *D* = -1.87, p < 0.01; S4 Table). On the contrary, neutrality indices were non-significant in *Vac* and *Cf* metapopulation (S4 Table). The observed lower level of expected homozygosity in the *Ca-Ud* metapopulation (θ_H_ = 0.42; Fig 3) compared to the other two associations (θ_H_ = 1.71 for *Vac* and 0.67 for *Cf*) could be a result of low-frequency alleles left over from geographical expansion [87]. Dividing this genetic cluster into two groups according to the host-plant (to deduce the source of the neutrality departure) revealed that the observed expansion signal derives from the *U*. *dioica* association with a highly significant negative value of neutrality indices (*F*_*S*_ = -8.59, p < 0.001; *D* = -2.18, p < 0.001; S4 Table). Furthermore, genetic differentiation between the *Ca*- and *Ud*-affiliated *H*. *obsoletus* populations was low (*F*_ST_ = 0.25, p < 0.001), with 75% of the divergence deriving from the inner host-plant associated variance. A very low genetic polymorphism was detected in the genetic structure of the *C*. *foetida* metapopulation, while the *V*. *agnus-castus* association expressed the highest level of inner divergence (pairwise differences, θ_π_), corresponding to 90.5% (p < 0.001) of the genetic variation observed between the Montenegrin and Greek haplogroups.

Microsatellite analyses were performed on 702 individuals from 50 populations (S1 Appendix). Fifteen new alleles were observed compared to previous data [7] resulting in 162 detected alleles. All loci were polymorphic across all populations. The mean number alleles/locus across seven loci = 23, range: 16 (F84) − 30 (C147). Total of 28 (17%) detected alleles were private, occurring in one population only. Private alleles were recorded in each plant association: *C*. *foetida* = 2, *C*. *arvensis* = 6, *V*. *agnus-castus* = 9 and *U*. *dioica* = 11. The highest number of private alleles within a single sample population was 7, occurring within *V*. *agnus-castus* association (Pop36 and 37), while 11 (7%) were private relative to a host-plant association (*V*. *agnus-castus* = 5 and *U*. *dioica* = 6). No significant linkage disequilibrium was detected for any locus pair in any population after Bonferroni’s correction [81].

The mean null allele frequency over all loci and populations was 0.06 with the highest value detected on locus B82 (0.11), followed by E96 and G85 (0.10) and should not bias the following analyses. The frequencies of null alleles correlated with mtDNA divergence are as follows: *C*. *arvensis* and *U*. *dioica* had an approximate null allele frequency of 0.05 (microsatellites were developed from a *U*. *dioica* population; [76]), *V*. *agnus-castus* had 0.06, while *C*. *foetida* had one of 0.09.

Expected heterozygosity *H*_*E*_ was independent of sample size (0.0005 < R2 < 0.16, p > 0.05). Hereafter we treated this parameter as an unweighted estimate. The mean genetic diversity estimates *H*_*E*_ and *A*_*R*_ did not differ significantly among populations affiliated with *C*. *arvensis* (*H*_*E*_ = 0.777 and *A*_*R*_ = 4.567), *U*. *dioica* (*H*_*E*_ = 0.765 and *A*_*R*_ = 4.455) and *V*. *agnus-castus* (*H*_*E*_ = 0.785 and *A*_*R*_ = 4.523), all p > 0.05 (Table 2). In contrast, the mean genetic diversity was significantly lower in the *H*. *obsoletus* populations associated with *C*. *foetida* (p < 0.05) than in the other host-associations (Table 2; *H*_*E*_ = 0.651 and *A*_*R*_ = 3.449). The observed signal is attributed to the Serbian, Romanian and Bulgarian populations, while the Turkish population had much higher values of diversity indices comparing to the European metapopulation (*H*_*E*_ = 0.768 and *A*_*R*_ = 4.379; Table 1).

**Table 2:** Comparison of microsatellite-based mean genetic diversity (*A*_*R*_—allelic richness and *H*_*E*_—expected heterozygosity) among four host-plant associated *Hyalesthes obsoletus* metapopulations.

	*C*. *arvensis*	*U*. *dioica*	*C*. *foetida*	*V*. *agnus-castus*
Mean *H*_*E*_	0.777	0.765	0.651*	0.785
Mean *A*_*R*_	4.567	4.455	3.449*	4.523
No of populations	9	20	13	8

* p < 0.05

*C*. *foetida vs C*. *arvensis H*_*E*_: t = 5.885, df = 20, p < 0.05; *A*_*R*_: t = 7.910, df = 20, p < 0.05

*C*. *foetida vs U*. *dioica*, *H*_*E*_: t = 5.355, df = 31, p < 0.05; *A*_*R*_: t = 6.945, df = 31, p < 0.05

*C*. *foetida vs V*. *agnus-castus*, *H*_*E*_: t = -5.291, df = 19, p < 0.05; *A*_*R*_: t = - 5.935, df = 19, p < 0.05

### Evolutionary relatedness among *Hyalesthes obsoletus* host-associated lineages

**Fig 4:**
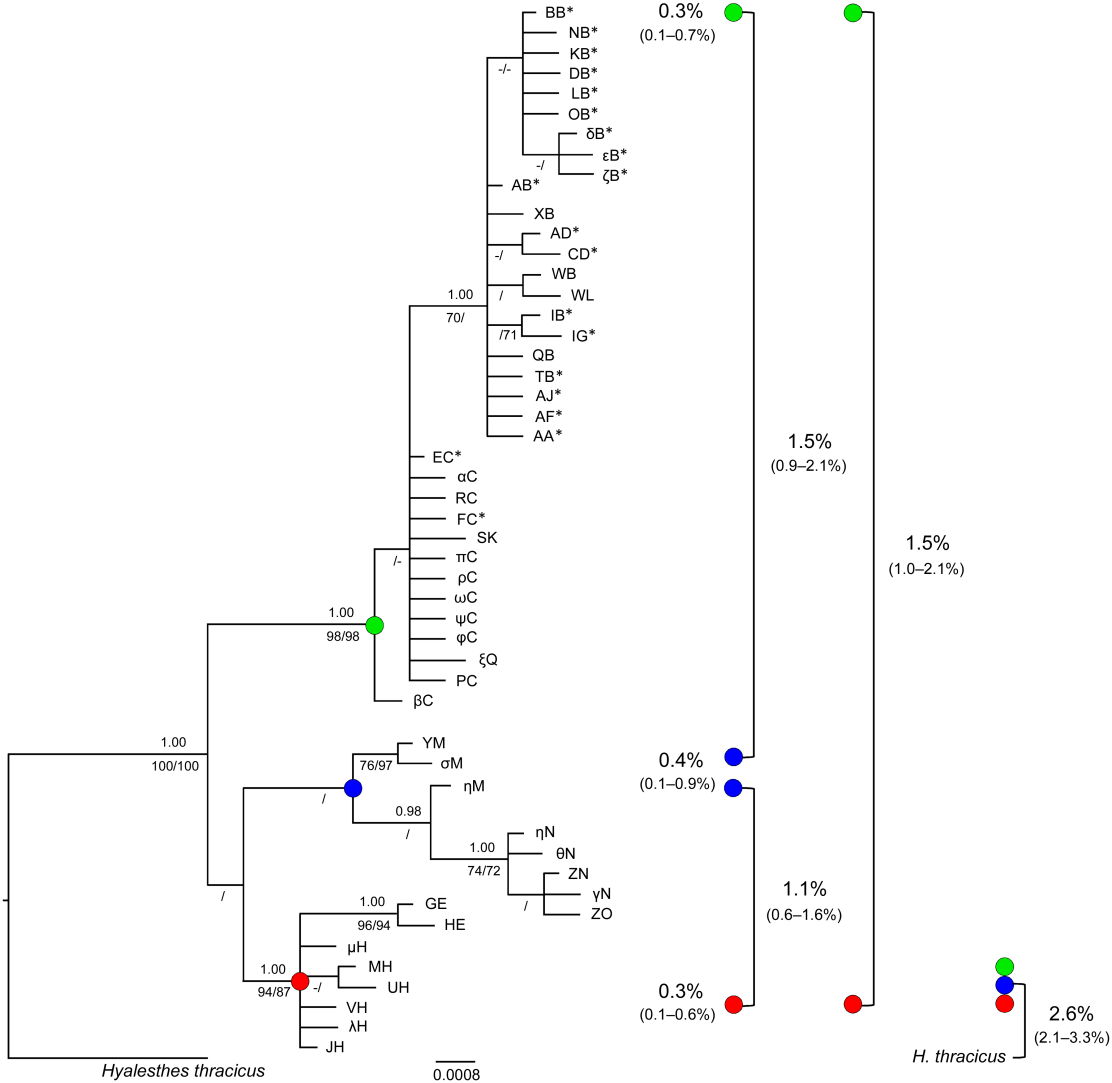
Bayesian phylogenetic tree inferred from 1180 bp of the *COI-tRNA(Leu)-COII* and *16S-tRNA(Leu)-ND1* concatenated mitochondrial gene regions sampled from the *Hyalesthes obsoletus* host-associated populations in this and previous studies. The Bayesian posterior probabilities are noted above branches, and the maximum parsimony and neighbor-joining bootstrap support values appear below the branches in that order. Bootstrap values below 70% and posterior probabilities below 0.95 are omitted. The nonrecovered nodes are marked with an em dash "–". The main nodes are designated with colored circles corresponding to host-plant associated haplogroups as follows: green = *Convolvulus arvensis* and *Urtica dioica*, blue = *Vitex agnus-castus*, and red = *Crepis foetida*. Average within-group genetic distances for each of the three host-plant clusters are noted left of the corresponding colored circle, while between-group distances are given on the right. The range of within- and between-group distances is given in parentheses. Distances are calculated with correction by applying the HKY+I nucleotide substitution model (Hasegawa-Kishino-Yano, pinvar = 0.881) among three genotype groups for all 51 haplotypes according to the three major host-associated clusters; therefore, the Israeli GE and HE haplotypes are considered as *Crepis foetida*-cluster members, while the QB, ψC and PC collected on crop plant are members of the *Convolvulus*-*Urtica* group. Distances between each *H*. *obsoletus* host-associated haplogroup and *H*. *thracicus* used as the outgroup are presented in the figure’s bottom right corner. Haplotypes detected in previous studies [8, 11, 15, 37] are marked with an asterisk (*).

A total of 51 host-associated *H*. *obsoletus* haplotypes were used for constructing a phylogenetic tree (Fig 4). Along with the previously noted 26 new haplotypes associated with the four host-plants detected in this study and the 22 previously described haplotypes associated with *Ca*, *Ud* or *Vac* [8, 11, 15, 37], phylogenetic analyses were supplemented with the data on the three new haplotypes (QB, ΦC, PC) collected from crop plants in Russia and Romania [7]. The Bayesian information criterion revealed the HKY+I (Hasegawa-Kishino-Yano, pinvar = 0.881) [88] to be the best substitution model for the ingroup sequences. This model was employed to estimate the pairwise genetic distances for the neighbor-joining tree and Bayesian analyses.

The obtained tree’s topology revealed a clear segregation of the *H*. *obsoletus* haplotypes into three phylogenetic clusters, each associated with host-plant preference: *Ca-Ud*, *Vac* and *Cf* (marked in green, blue and red in Fig 4, respectively). All three phylogenetic analyses resulted in the same general tree topology. Monophyly of the *Ca-Ud* and *Cf* mitochondrial lineages was supported by high Bayesian posterior probabilities (1.00) and bootstrap support values (87–98%), while values for the *Vac* lineage were lower (0.86, 53% and 56%) probably due to geographic diversity. In addition, the *Vac* and *Cf* lineages formed a joint branch, but with low support values (0.52, 53% and 58%) due to high inner-branch diversity. However, the bifurcations were supported using genealogical network analysis (see below).

**Fig 5:**
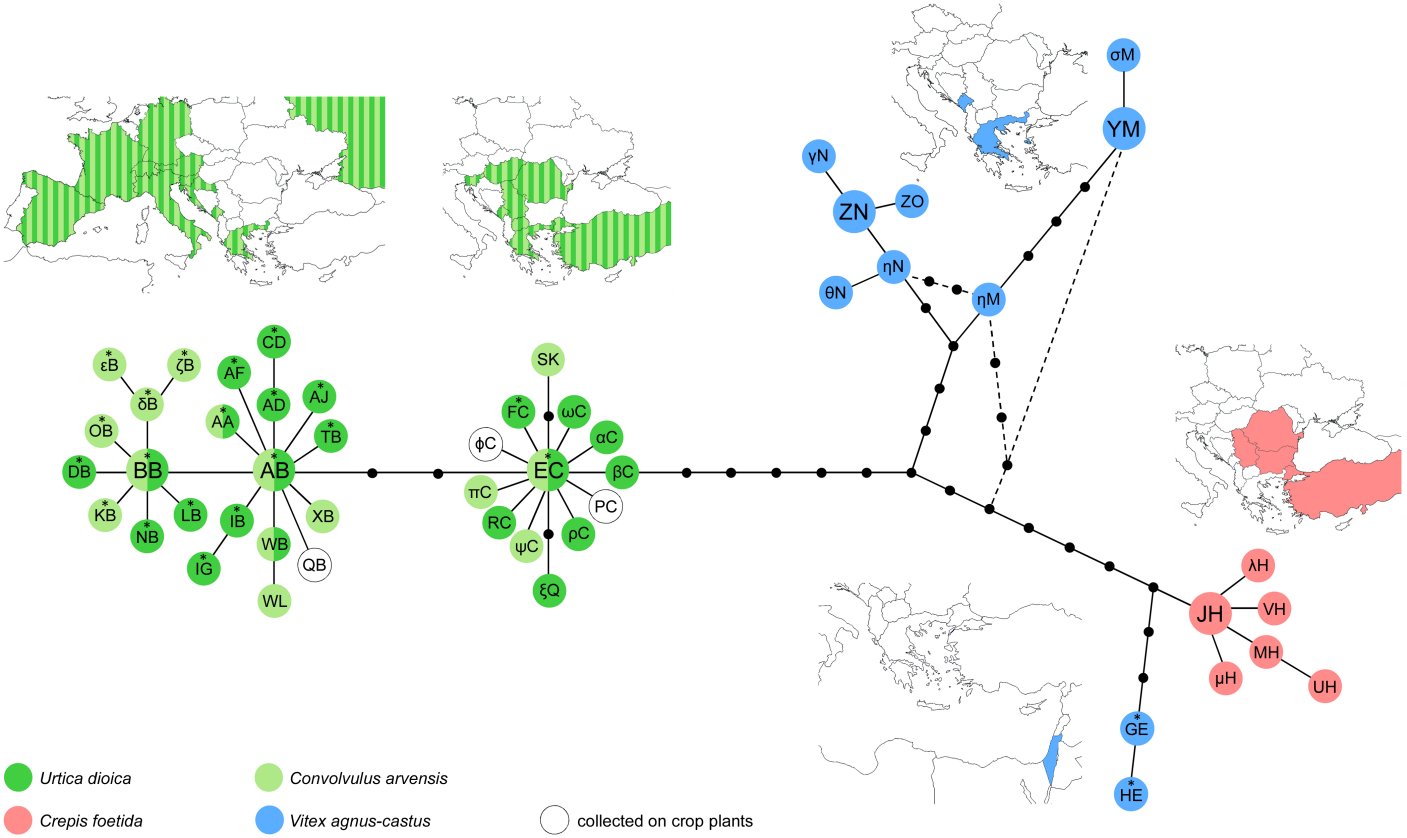
The phylogenetic haplotype network obtained using median-joining and statistical parsimony algorithms on concatenated *COI-tRNA(Leu)-COII* and *16S-tRNA(Leu)-ND1* mitochondrial gene regions of the 51 *Hyalesthes obsoletus* haplotypes identified in this and previous studies. Haplotype colors correspond to the host-plant associations. Haplotypes detected in previous studies [8, 11, 15, 37] are marked with an asterisk (*). The most common haplotypes within each haplogroup are noted with enlarged circles. Dashed lines represent alternative variants of network formations obtained using both algorithms. Black dot vertices represent missing or unsampled haplotypes. Distribution maps are given above each host-associated *H*. *obsoletus* haplogroup. Each detected haplogroup’s country is designated on maps in the color corresponding to the associated host-plant. Because *Convolvulus arvensis* and *Urtica dioica* share a number of *H*. *obsoletus* haplotypes, BB-AB and EC haplogroup distribution is designated in two shades. Haplotypes detected in the two previously reported *H*. *obsoletus* populations collected on crop plants in Romania and Russia (Radovanu and Mayak) [7] are not colored.

The mitochondrial genealogical network revealed a combination of clear and ambiguous host-plant affiliations, as well as geographic divisions within host-plant associated *H*. *obsoletus* haplotypes (Fig 5). Two alternative networks were obtained using both algorithms; however, these networks differed only in the bifurcation position of the major three branches (dashed lines in Fig 5). The populations affiliated with *C*. *arvensis* and *U*. *dioica* share five haplotypes and have ten and 17 private ones, respectively. They were separated into two phylogeographic sub-clades: a Western European haplogroup (BB- and AB-derived haplotypes) and an Eastern European haplogroup (EC-derived haplotypes). An exception to this geographic based distribution was the Russian haplotype QB, which belonged to the western haplogroup. It implicates a common host-plant association older than the phylogeographic division. *V*. *agnus-castus* haplotypes were paraphyletic, being divided into three subgroups consisting of the monophyletic Greek YM and Montenegrin ZN haplotype clades (three and five haplotypes, respectively) and a paraphyletic Israeli clade (two haplotypes). The two Israeli *Vac* haplotypes (GE and HE) were positioned closest to the *C*. *foetida* network branch as well as in the evolutionary tree (Fig 4). The six haplotypes affiliated with *C*. *foetida* were delimited as a monophyletic clade, all deriving from the JH haplotype. JH was detected in all *Cf*-associated *H*. *obsoletus* populations, which were collected over a wide geographic range, from east Turkey to east Serbia.

The average pairwise genetic divergence between the three host-plant associated phylogenetic clusters varied between 1.1% and 1.5%, while the variance within clusters was 0.3–0.4% (Fig 4). Divergence estimates based only on the phylogenetically informative *COI-COII* gene region (711 bp) were higher, 2.5% (S5 Table). The genetic divergence between host-plant associated phylogenetic clusters and *H*. *thracicus*, the most closely related valid species in the species group, varied between 2.1% and 3.3% for the entire analyzed mtDNA segment and 2.9–3.8% for the *COI-COII* region (Fig 4, S5 Table). Thus, genetic distances among the three clusters of host-associated *H*. *obsoletus* haplotypes, on the one hand, and between *H*. *thracicus* as a morphologically delineated species on the other, suggested evolutionary relationships of three closely related, but separate, cryptic species delineated by host-plant choice.

### Host-associated population differentiation: mitochondrial data

**Fig 6:**
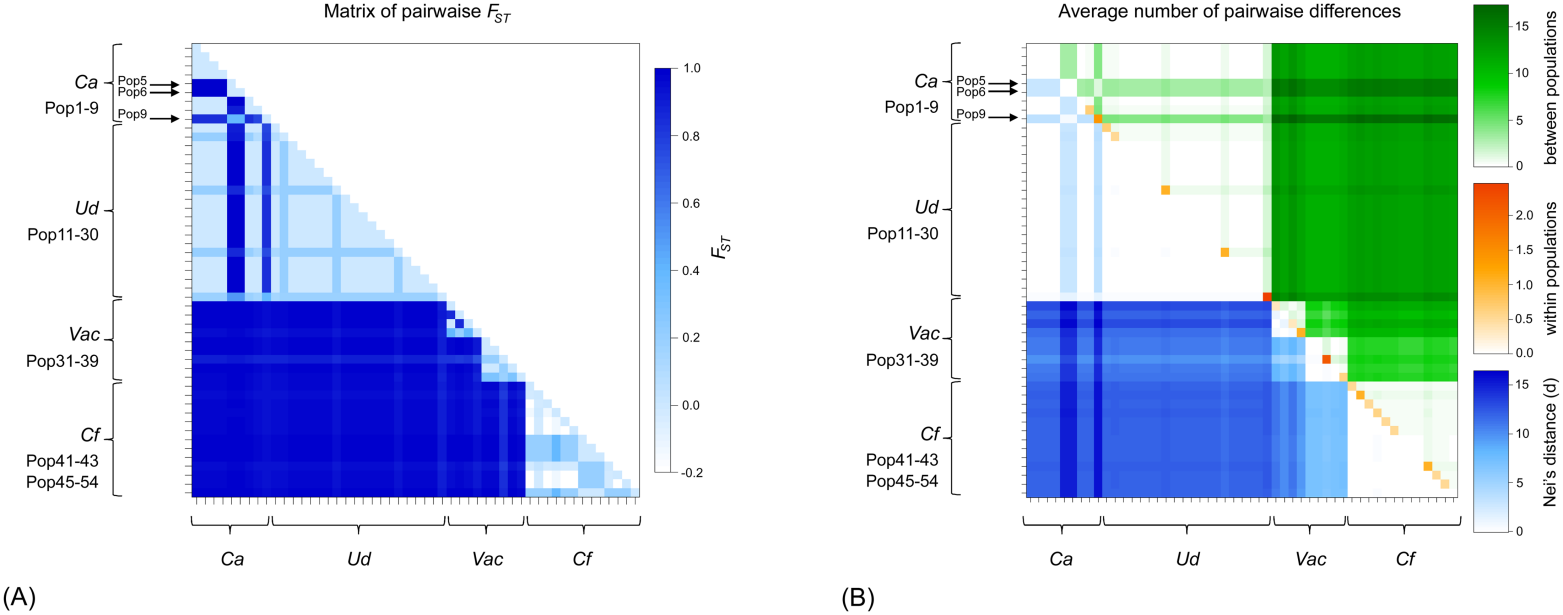
Plots representing parameters of mtDNA genetic differentiation between *Hyalesthes obsoletus* host-associated populations based on (A) pairwise *F*_*ST*_ values and (B) Nei’s average number of pairwise differences. Associated host-plants: *Convolvulus arvensis* (*Ca*), *Urtica dioica* (*Ud*), *Vitex agnus-castus* (*Vac*), and *Crepis foetida* (*Cf*). Populations are listed in the same order as in Table 1. The three populations marked with arrows show genetic differentiation compared to all others in the same host-associated group (*Ca*-*Ud*). The blue elements below the diagonal of *F*_*ST*_ values range from 0 to 1, with 0 (including < 0) indicating no divergence between the populations and 1 indicating that two populations are completely separated. The green elements above the diagonal denote Nei’s average number of pairwise differences among populations, the orange diagonal elements denote Nei’s average number of pairwise differences within populations, and blue elements below the diagonal denote the net number of nucleotide differences among populations (Nei’s distance).

The measure of pairwise population differentiation due to genetic structure (*F*_ST_) as well as Nei’s pairwise differences among and within populations clearly showed the existence of three genetically separate groups associated with different host-plants: 1) *C*. *arvensis* and *U*. *dioica*, 2) *V*. *agnus-castus* and 3) *C*. *foetida* (Fig 6). Additionally, the genetic structure analysis within associations revealed further population differentiation between some of the populations in the *Ca-Ud* group (Pop 5, 6 and 9) and within the *Vac* group.

Genetic differentiation among the 51 host-associated *H*. *obsoletus* populations provided by the *F*_ST_ values were high (from 0.81 to 1.00) and significant (p < 0.01) when the populations associated with *Ca*-*Ud*, *Vac* and *Cf* were compared to each other (Fig 6; dark blue and green elements on plots). Conversely, the differentiation among populations associated with *Cf*, including the geographically distant population from Erzincan in east Turkey, was always low, *F*_ST_ = 0.00 to 0.20 (Fig 6). Within the population group associated with *Vac* populations originating from Montenegro and Greece were highly differentiated (*F*_ST_ = 0.73–1.00; p < 0.01), while differentiation among the Greek populations was low (*F*_ST_ = 0.00–0.30). Differentiation among most *Ca* and *Ud* population, regardless of geographic origin, was low, *F*_ST_ = 0.00 to 0.20. Nevertheless, three populations associated with *Ca* in Montenegro and Greece (Pop5, 6 and 9; marked with arrows in Fig 6) showed high and significant differentiation from the other populations within the *Ca*-*Ud* associations (*F*_ST_ = 0.70–1.00; p < 0.01). This finding was caused by the presence of the AB-group haplotypes in these populations (Table 1), while EC-group haplotypes were found in the other *Ca-Ud* populations. The genetic differentiation of these three populations was less prominent (*F*_ST_ = 0.40–0.50, p < 0.05) only in comparison with the *Ud*-associated Pop30 (Profitis, Greece) because of the mixed presence of the AB- and EC-group haplotypes in this population.

Estimates of Nei’s pairwise mean genetic distances among populations confirmed differentiation in relation to the host-plant (Fig 6B). The maximum pairwise difference was detected between the *Ca-Ud* and the *Cf*-associated populations (12–15) as well as between the *Ca-Ud* and *Vac*-associated populations (9–12). Within host-plants, pairwise genetic distances were 0.0–4.0 for the *Ca-Ud* associated populations, 0.0–1.2 for the *Vac*-associated populations in Montenegro and 0.0–2.1 in Greece (4–8 in between), and 0.0–1.0 for the *Cf*-associated populations. The within-population genetic distances were found for those associated with *Cf*, and were caused by the number and frequency of haplotypes (Fig 6B, orange elements).

In syntopy, i.e., co-occurrence of two host-plants harboring *H*. *obsoletus* (11 localities; Table 1), mtDNA haplotypes were always affiliated to the *a priori* defined host-plant association: AB or EC haplotype group with *Ca* or *Ud*, ZN or YM with *Vac* and JH associated with *Cf*. These findings unequivocally confirmed haplotype (lineage) specificity to host-plant.

### Evidence of host-associated population differentiation: Microsatellite data

**Fig 7:**
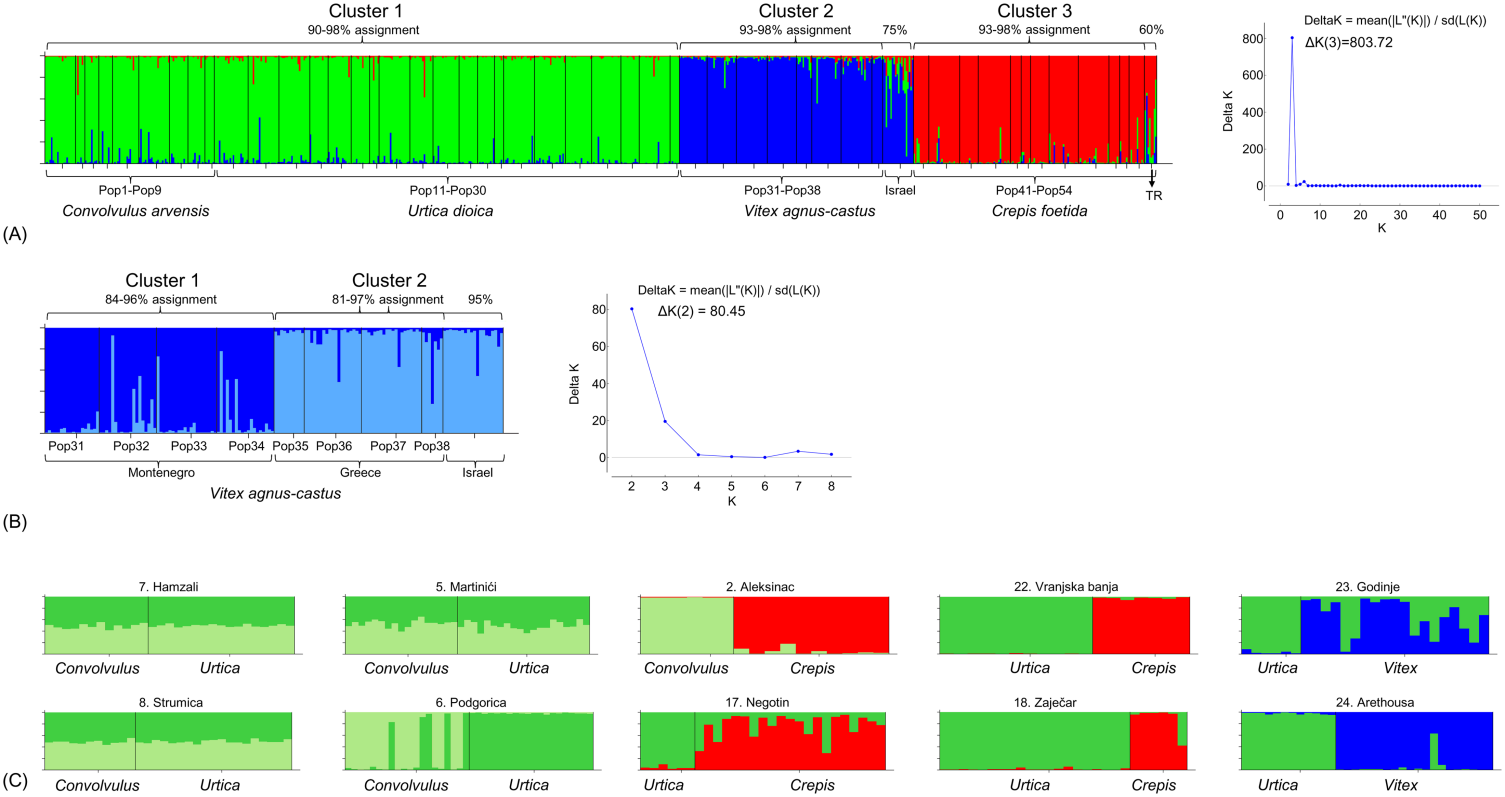
Bar plots of the Bayesian clustering analysis performed using Structure software on microsatellite data. (A) 702 *Hyalesthes obsoletus* individuals from the 50 populations genotyped in this study and a single 20-member population associated with *Vitex agnus-castus* from Israel [7], suggesting a ΔK = 3 as the most likely number of genetic clusters; (B) 132 *H*. *obsoletus* individuals from the 8 populations associated with *Vitex agnus-castus* in Montenegro and Greece genotyped in this study and the aforementioned Israeli population [7], suggesting a ΔK = 2 as the most likely number of clusters; and (C) the ten syntopic localities of the *H*. *obsoletus* populations associated with two host-plants. Each column on the plots represents a single individual and the vertical black lines divide individuals by population. Colors represent proportional membership in each genetic cluster (green = *Convolvulus arvensis* and *Urtica dioica*, blue = *Vitex agnus-castus*, and red = *Crepis foetida*). Population membership assignments to the suggested clusters are designated above bar plots (A) and (B).

Bayesian clustering analysis performed with Structure based on the 50 *H*. *obsoletus* populations (702 individuals) from southeastern Europe and Turkey, and a single *Vac*-associated population from Israel (20 individuals) [7], supported the existence of three genetic groups (Evanno’s ΔK(3) = 803.72). The average genetic membership probabilities were > 90% and clustered as follows: the *Ca*- and *Ud*-associated individuals formed Cluster 1, *Vac* association formed Cluster 2, and the *Cf*-associated individuals formed Cluster 3 (Fig 7A), thus corroborating the mtDNA analysis results. The microsatellite analysis clustered the Israel *Vac* population with the Balkan *Vac* populations. This result contrasts the mtDNA analysis where these individuals cluster closer to the *Cf* metapopulation (Figs 4 and 5). However, the Israel population had a lower membership assignment of 75% to the *Vac* host-plant group in the overall analysis (Fig 7A). Estimates of molecular genetic variance (Amova) showed that 77% of the total genetic variance was host-plant affiliated (S6 Table). To elucidate the substructure of the three clusters, we analyzed each of them separately. Neither Cluster 1 (*Ca* and *Ud*, 29 population ΔK(2–28) = 0.06–11.14) nor Cluster 3 (*Cf*, 13 populations ΔK(2–12) = 0.18–5.32) showed signals of geographic differentiation, and for *Ca* and *Ud* there was no significant host-plant differentiation. In contrast, genetic variance in Cluster 2 (*Vac*) was divided between Montenegrin and the Greek metapopulations (approx. 400 km), geography explaining 60% of the total variance (S6 Table). The Structure analysis of all *V*. *agnus-castus*-related populations grouped Israel and Greek populations to the same subcluster (ΔK(2) = 80.45; Fig 7B) with 81–97% population membership assignment. An Amova of Montenegrin vs. Israel and Greek populations estimated 42% of the total genetic variance was caused by differentiation between the two clusters (S6 Table).

Among 11 syntopic sites harboring two host-associated *H*. *obsoletus* populations, ten were analyzed for genetic differentiation based on microsatellite data (Fig 7C). The population from *C*. *arvensis* in east Turkey (locality Erzincan, Pop10), syntopic with the *C*. *foetida*-associated population (Pop54), was removed from the analyses because the number of collected individuals was low (3 specimens). In three of the four analyzed syntopic *Ca-Ud* sites (Hamzali, Martinići and Strumica) no differentiation between host associations was found, agreeing with the general absence of the host-driven diversification signal for all *Ca-Ud* populations. However, in one *Ca-Ud* syntopic site (Podgorica), Evanno’s ΔK(2) = 76.94 suggested the existence of two clusters, hence showing a signal similar of host races as in southwest Germany (Fig 7C). At this site phylogeographic-related western AB-group haplotypes were found in the *Ca*-associated population (Pop 6) and eastern EC-group haplotypes were found in the *Ud*-association (Pop25). In all other syntopic localities (*Ca*/*Cf*, *Ud*/*Cf* or *Ud*/*Va*c), clear population-based host-plant affiliation were evident (population membership asignment 68–99%), but not for all individuals of the affiliated host-plant (Fig 7C). MtDNA analysis of these “wrongly assigned” individuals confirmed them as members of the source host-plant mtDNA haplogroup; hence, a genetic discrepancy was exhibited between mtDNA and microsatellite data (Figs 6 and 7).

**Fig 8:**
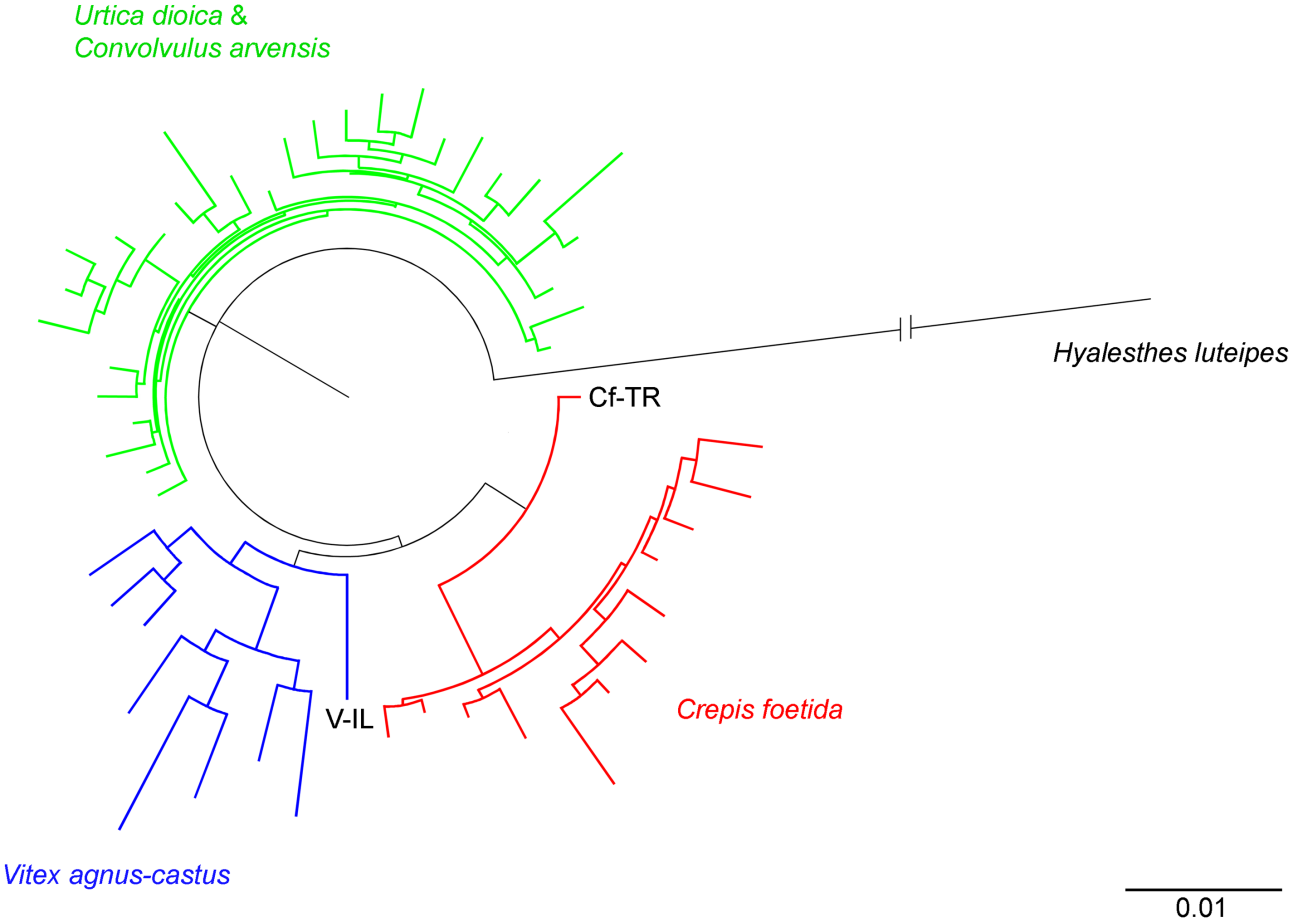
Maximum-likelihood phenogram based on allele frequencies calculated with four microsatellite loci for the 51 populations of *H*. *obsoletus* (722 individuals). Individuals were associated with *Convolvulus arvensis* and *Urtica dioica* (green branches), *Vitex agnus-castus* (blue branches) and *Crepis foetida* (red branches) from Southeastern Europe and Turkey analyzed in this study and for the Israeli population associated with *V*. *agnus-castus* [7]. *Hyalesthes luteipes* was used as an outgroup to root the tree. The two most divergent populations of the *Crepis foetida*-associated genotype group from east Turkey (Cf-TR) and the *Vitex agnus-castus*-associated genotype group from Israel (V-IL) are designated.

The maximum likelihood phenogram, based on 4 out of 7 microsatellite loci that amplified in the outgroup specimens of *Hyalesthes luteipes*, divided populations according to host-plant associated differentiation (Fig 8).

## Discussion

In recent years, several studies have documented specialized plant-associated life cycles of *Hyalesthes obsoletus* [7, 14, 15, 33, 38]. These observations lead Kosovac et al. [34] to hypothesize that *H*. *obsoletus* is a species complex undergoing cryptic speciation. In this paper, we analyzed genetic separation of four host-plant associated *H*. *obsoletus* populations in southeastern Europe, the ancestral distribution range of *H*. *obsoletus*. Our results clearly indicate three distinct genetic entities undergoing complex genetic diversification processes, and they question the taxonomic integrity of *H*. *obsoletus sensu lato*. Differentiation among three morphologically indistinguishable plant affiliated associations of *H*. *obsoletus*, 1) *C*. *arvensis* and *U*. *dioica*, 2) *V*. *agnus-castus* and 3) *C*. *foetida* show much higher segregation on an evolutionary time-scale compared with the previously identified *C*. *arvensis* and *U*. *dioica* host races in south Germany [7] or that suggested for *Salvia sclarea*- and *Lavandula angustifolia*-associated host-race formation in south France [11]. The following evidence for sibling species-level relatedness revealed in these host-plant relations are: i) clear and consistent mitochondrial genealogical divergence between host-associated populations in both syntopy and across the geographic distribution range (i.e. in allopatry); ii) nuclear genetic divergence supporting mitochondrial genealogy; and iii) the average genetic distance among mitochondrial haplotypes of host-associated populations (1.1–1.5%) is comparable to that of the most closely related morphologically separated species, i.e., *Hyalesthes thracicus* (2.1–3.3%).

### Three cryptic genetic lineages deriving from four host-plant associations

This study was initially started as a survey to verify *H*. *obsoletus*’ affiliation with *C*. *arvensis* and *U*. *dioica*, the traditional host plants in southeastern Europe. Relations between these two associations were expected to be complex. Evidence for host races in the western European mtDNA sub-haplolineage (AB) was found in an area of recent immigration in Germany and northern France, but not in Western Europe south thereof [7, 15, 28]. The present study from southeastern Europe, where most *Ca-Ud*-associated individuals belong to the EC mtDNA sub-haplolineage also suggest a lack of host races associated with *C*. *arvensis* and *U*. *dioica* there. Combined data indicate an innate capability of *H*. *obsoletus* to utilize both plants in the ancestral distribution range. However, the finding of the core populations’ single syntopic locality with clear genetic differentiation on the host-plant level (Pop6 and 25, Podgorica, Montenegro), require further investigation. One reason for differentiation here may be phylogeographic bias rather than host-plant specialization. Individuals associated with the two plants belonged to the sub-lineages AB and EC, respectively. These sub-lineages are also genetically diverged for microsatellite loci [8].

The association with *V*. *agnus-castus* was described by Hoch and Remane [31] and need not have been a novelty for Europe. However, researchers have focused on preferences for crop weeds rather than its known wild-plant associations [31]. Prior to our study, the only recent data pointing to this plant association were from Israel, where it was experimentally confirmed as a true host [33]. In our study, we detected associations of *H*. *obsoletus* with *V*. *agnus-castus* in Montenegro and Greece (mainland and islands) and we believe that the association will be affirmed along the coast of the Adriatic Sea, as well as in other parts of the Mediterranean basin—such as the coastal regions of the Iberian, Apennine and Anatolian peninsula—as outlined in Hoch and Remane [31]. Recently, the *V*. *agnus-castus* association was confirmed in habitats surrounding vineyards along Bosnia and Herzegovina’s coastal area [89], while its occurrence in North Dalmatia (Croatia) was described in the late 19th century [32], but since forgotten. According to our data, *H*. *obsoletus* associated with *V*. *agnus-castus* on the Balkan Peninsula consists of two geographically separated genotype groups (Greek YM and Montenegrin ZN), which are linked genealogically by the haplotype ηM. The spatial separation between the groups is approximately 250 km along the coastline with suitable habitats for *V*. *agnus-castus* growth. Whether the two *V*. *agnus-castus* sub-lineages of *H*. *obsoletus* represent two subspecies separated by the Pindus mountain range as a barrier that prevents between-group interaction, or simply are phylogeographic variants, may be assessed by sampling in the intermediate range along the Albanian coastline.

One of the most interesting findings is a closer mtDNA genealogical relatedness of Israeli *V*. *agnus-castus* haplotypes to the *C*. *foetida*-associated haplo-group than to the Montenegrin and Greek haplotypes (Figs 4 and 5). Conversely, the Bayesian clustering analysis of nuclear data identified Greek and Israel *V*. *agnus-castus* specimens as monophyletic. Hence, a host shift and further diversification between populations associated with these two hosts has probably occurred in the area of Middle East; however the direction of the shift can only be speculated due to insufficient data. The host shift of a phytophagous insect to a new plant is a demanding, mainly because of the phytochemical barriers [90]. Populations that have undergone host shift could use ancestral and new host plants as a niche, but they might not be able to use them simultaneously, a phenomenon that can be detected by a host-plant associated fitness trade-offs on the ancestral host [2, 90, 91]. Therefore, it could be assumed the collection of specimens that are affiliated with one host-plant by preferred plant choice (e.g., *V*. *agnus-castus*), but that are linked to other association according to mtDNA genetic markers (i.e., *C*. *foetida*), as a relic from their host-shift past.

An unexpected result in this study was the genetic uniqueness and affiliation of *H*. *obsoletus* populations to *C*. *foetida* across a vast area ranging from east Turkey up to the Danube and Morava rivers on the Balkans (Fig 1). Although we have only few samples from Anatolia, some interesting and important findings have arisen because these specimens occur toward the eastern and southern borders of the *H*. *obsoletus* distribution. Güclü and Ozbek [92] reported the association with *C*. *arvensis* in Erzurum; we confirmed the association in 150-km distant Erzincan in eastern Turkey. The *C*. *foetida* association was encountered in Kırşehir and syntopically with *C*. *arvensis* in Erzincan. Much genetic specificity can be attributed to this host-plant association which indicates a detachment of this metapopulation from the rest of the *H*. *obsoletus* s.l. scope. A low number of private alleles indicates the tightness of this association and the low number of migrants [93] as well as the decrease in allelic richness of the European populations imply a drifting departure from the Middle Eastern ancestral area, contributing to the *H*. *obsoletus* hypothesized Levantine origin [15]. Meanwhile lower heterozygosity values, compared with other two associations, can be attributed to discrepancy forces such as inbreeding and genetic drift, probably due to westward range expansion. The eastward perimeter of the *C*. *foetida*-associated distribution is currently unknown, but according to available data, it expands to Erzincan in northeast Turkey, while westward we assume that Danube and Morava rivers are barriers toward central Europe, although a single population north of the Danube was recorded (Romania, locality 42). This association could have entered the European continent by crossing the Bosphorus and the Dardanelles or by following the Danube valley, like many other taxa that share this Balkan-Anatolian and Ponto-Panonian chorotype [94]. Haplotypes associated with *C*. *foetida* collected on crop plants in Radovanu (south Romania) and the presence of this association on both sides of the Danube in Bulgaria and Romania (Fig 1) support a Danube expansion rout.

### Cryptic speciation in *Hyalesthes obsoletus*: A need for an integrative taxonomic approach

Due to its significance as a vector of plant pathogenic phytoplasma, *H*. *obsoletus* is the most well known representative of the south Palaearctic planthopper genus *Hyalesthes*. However, as the present study shows, its specific, plant-associated lineages moreover raise significant concern in the context of integrative taxonomy. This is further emphasized by fact that *H*. obsoletus is the type species, i.e., the name bearing type of the genus. Efforts made to locate *H*. *obsoletus* holotype specimen have failed [31], leading to the general conclusion that it is probably lost. However, according to the original description [95], the type specimen was collected in south France "France mérid. (Grenier), Chambéry (Cartereau)" as the type-locality, but a description of the host-plant is lacking. In addition, type material of a single *H*. *obsoletus* synonym, *Liorhinus albolimbatus* Kirchbaum, 1868 (synonymized by Fieber [96]) collected in Dalmatia is also probably lost [31]. Hence, attributing a type specimen to any plant association would be speculative, but knowing that all four wild host-plants are present in the vicinity of the type localities (http://ww2.bgbm.org/EuroPlusMed/query.asp) emphasizes a need for genetic analysis of this archival material (if located) and its mere importance for resolving *H*. *obsoletus* taxonomic and nomenclature status.

Hoch and Remane’s revision of genus *Hyalesthes* [31] was based on rich entomological collections. Cladistic analysis based on morphological characters revealed the existence of 33 species belonging to five monophyletic species groups [31, 36, 54, 97, 98]. The great morphological similarities among species within the same species group suggest a very shallow evolutionary history of these taxa. However, all within-group species can be reliably differentiated based on male genital armature [31], including *H*. *obsoletus* and its closest relative *H*. *thracicus*. These subtle phenotypic differences, and an average genetic distance of 2.6% (3.3% on *COI-COII*), suggest a recent common ancestor not older than the Quaternary glacial cycles [99–101]. The Mid-Atlantic Islands (Canaries and Madeira) and the east Mediterranean (Turkey-Anatolia) are two diversity centres of the genus *Hyalesthes* [54, 102]. Our genetic data indicate, in accordance with Hoch and Remane [31], an origin of *H*. *obsoletus* in the eastern Mediterranean. Micro-refugia as well as host-plant specialization likely expanded the distribution range of *H*. *obsoletus* westwards (Fig 5).

Haplotype divergence among the three host-plant associated *H*. *obsoletus* meta-populations was similar (ca. 2%) but morphological differences were not evident among them (A. Kosovac, J. Jović and I. Toševski, unpublished result). This lack of morphological difference may imply intense specialization to specific plant metabolites that rapidly separated the incipient species. Berlocher and Feder [17] argued for an incipient stage of sympatric speciation and host-associated species as the differentiation climax where populations are considered “operational species” when significant genetic, behavioral and morphological differences are present at two sympatric localities. Following the definition of sympatric speciation that implies the divergence of one evolutionary lineage into two in the absence of geographic isolation, we consider morphological delineation as the last step before true species status. In our study, we detected seven syntopic sites (two *Ca*/*Cf*, three *Ud*/*Cf* and two *Ud*/*Vac*) where plant-associated populations lacked genetic exchange. Hence, we view *H*. *obsoletus* as a species complex, for which cladogenesis is linked to morphological stasis [103]. The decoupling of genetic and morphological divergence is common for young cryptic species where morphological traits or other diagnosable features have not yet evolved [103, 104].

Following genetic and ecological (host-plant) differentiation of three entities within, what is up-to-date considered a single unique species under the name *H*. *obsoletus*, requires an integrative taxonomic approach. If the type material of *H*. *obsoletus* is considered lost, then the designation of a neotype, following the International code of Zoological nomenclature (ICZN), can lead to stabilization of the taxonomic and nomenclature ambiguity within the *H*. *obsoletus* species complex.

### Influence of vectors’ divergence on stolbur phytoplasma epidemiology

Clarifying the true relationships of host associations of insect vectors is of particular epidemiological importance because these associations determine the breadth of pathogen transmission. For *H*. *obsoletus*, which acquires stolbur phytoplasma in the larval stage [24], knowledge of dual hosts is paramount. Disease cycles, which may or may not overlap, depend on the vectors’ plant preference [13, 14, 37]. Our results show that two opposing phenomena determine the epidemiology of stolbur phytoplasma vectored by *H*. *obsoletus*. First, the genetic homogeneity of *H*. *obsoletus* utilizing *C*. *arvensis* and *U*. *dioica* is opposed by two distinct plant associations of the pathogen, *tuf*-b and *tuf*-a, respectively [14]. Second, the *V*. *agnus-castus* and *C*. *foetida* cryptic species transmit only *tuf*-b [13, 38, 40]. Hence, epidemiological cycles are partly "un-coupled" relative to genetic diverged host-races or cryptic species and their host-plant associations. The multilocus genotypization of the pathogen strains in cross-transmission by naturally infected populations affiliated with *C*. *arvensis*, *V*. *agnus-castus* and *C*. *foetida* indicate the existence of intersections or meeting points in the epidemiological pathways of *tuf*-b strains associated with different host-plants and plant-specialized vector populations (A. Kosovac and J. Jović, unpublished data) [40]. However, considering the vector’s genetic structure it is more likely that when shared phytoplasma genotypes appear at meeting points in dual host-plants they are a consequence of feeding (i.e. transmission) by other vectors of minor or intermediate importance [9, 10, 13], than a result of *H*. *obsoletus* polyphagy. Although we do not exclude the possibility that *H*. *obsoletus* belonging to a specific association (e.g. *C*. *arvensis*) feeds on erroneous plants of another association (e.g. *V*. *agnus-castus*) within the same epidemiological cycle (*tuf*-b), this event is unlikely in many habitats of the species range due to differences in micro-geography, micro-climate and vegetation composition. Survival tests of genetic host-races on alternative plants [29] show that *H*. *obsoletus* is more dependent on the host-plant than was previously thought. Hence, host-plant preference, which is proven to be a crucially important trait for this insect, requires special attention in all further studies delimiting epidemiological cycles. Therefore, to verify that plants currently found in the literature are actual hosts for development, we encourage careful population sampling in the entire distribution range and molecular confirmation of larval presence on the plants’ roots to determine true host suitability and association. Only precise identification of dual hosts enables detecting pathogen transmission routes and epidemiological cycles for relevant management strategies, e.g., [13].

This thrilling evolutionary story of *H*. *obsoletus* occurs in natural ecosystems, unlike the majority of economically important insects, for which cryptic differentiation is usually attributed to survival and reproductive pressures in agro-ecosystems, e.g., [105–107]. The study’s insights into cryptic divergence of *H*. *obsoletus* support the notion that specialization and possibly cryptic species is more common than often appreciated in "generalist" insects [108]. It poses a challenge to taxonomic delimitation; erroneously delineating pest species has significant implications for success of short- or long-term control management.

## Supporting information

S1 Appendix Raw microsatellite data for 50 *H*. *obsoletus* population. (TXT)

S1 Fig Archival museum specimen of *Hyalesthes thracicus* Hoch, 1986 paratype used as outgroup for phylogenetic analyses of *Hyalesthes obsoletus* host-associated haplotype-groups. (PDF)

S1 Table Literature data overview on wild and cultivated plants associated with *Hyalesthes obsoletus* feeding, aggregation and/or development resulting in polyphagous perception of this vector. (PDF)

S2 Table Primers used for amplification of mitochondrial *COI-tRNA(Leu)-COII* and *16S-tRNA(Leu)-ND1* gene regions in *Hyalesthes obsoletus* and archival paratype specimen of *H*. *thracicus*. (PDF)

S3 Table List of *Hyalesthes obsoletus* newly identified haplotypes of the two mtDNA gene regions with corresponding GenBank accession numbers and data on host-plant associations. (PDF)

S4 Table Neutrality tests for the *Hyalesthes obsoletus* host-plant grouped populations and the overall dataset based on mtDNA *COI-tRNA(Leu)-COII* and *16S-tRNA(Leu)-ND1* data. (PDF)

S5 Table Average evolutionary distances calculated for the *COI*-*COII* gene region (711 bp) for 37 *H*. *obsoletus* haplotypes (excluding the Q, Φ and P, and the Israeli G and H derived haplotypes), corrected by applying the HKY+I substitution model. (PDF)

S6 Table AMOVA *F*-statistical comparison of genetic divergence within and between *Hyalesthes obsoletus* populations grouped according to host-plant associations or geography. (PDF)
