# Supplementary material for: Widespread plant specialization in the polyphagous planthopper Hyalesthes obsoletus (Cixiidae), a major vector of stolbur phytoplasma: Evidence of cryptic speciation
Source: PLoS One. 2018 May 8;13(5):e0196969. doi: 10.1371/journal.pone.0196969 (PMC5940214; doi:10.1371/journal.pone.0196969)
Supplement: S1 Fig — (PDF) [file pone.0196969.s002.pdf]

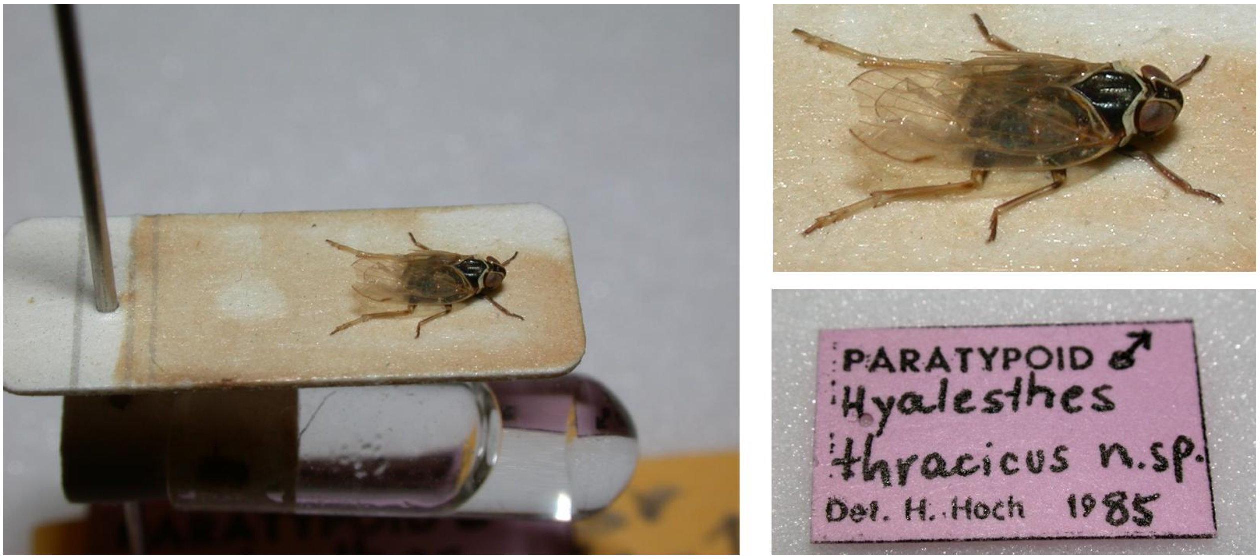

S1 Fig. Archival museum specimen of *Hyalesthes thracicus* Hoch, 1986 paratype used as outgroup for phylogenetic analyses of *Hyalesthes obsoletus* host-associated haplotype-groups. The specimen's mitochondrial DNA was analyzed using specifically designed primers for amplification of short DNA fragments (Fig 2, Table S2). The specimen originated form Prof. H. Hoch's private collection, now housed at the Institute for Plant Protection and Environment collection (IPPE, Zemun, Serbia).
