# Supplementary material for: Widespread plant specialization in the polyphagous planthopper Hyalesthes obsoletus (Cixiidae), a major vector of stolbur phytoplasma: Evidence of cryptic speciation
Source: PLoS One. 2018 May 8;13(5):e0196969. doi: 10.1371/journal.pone.0196969 (PMC5940214; doi:10.1371/journal.pone.0196969)
Supplement: S1 Table — (PDF) [file pone.0196969.s003.pdf]

S1 Table Literature data overview on wild and cultivated plants associated with *Hyalesthes obsoletus* feeding, aggregation and/or development resulting in polyphagous perception of this vector.

Country	Plant	Note	Reference
Russia	*Convolvulus arvensis*<br>*Lepidium draba*	nymphs and adults	Sukhov & Razvyazkina 1955
	Solanum tuberosum (potato)	adults	Imo *et al.* 2013
Turkey	*Cistus* sp.<br>*Crataegus* sp.<br>*Myrtus* sp.<br>*Paliurus* sp.<br>*Quercus coccifera*<br>*Quercus ilex*<br>*Rubus* sp.<br>*Sesamum indicum*<br>*Vitex* sp.<br>*Capsicum anuum* (paprika)	adults	Hoch & Remane 1985
	Convolvulus arvensis<br>*Crambe orientalis*<br>*Falcaria vulgaris*<br>*Geranium tuberosum*<br>*Isatis glauca*<br>*Lepidium draba*<br>*Melilotus officinalis*<br>*Onosuma armeneum*<br>*Rumex crispus*<br>*Taraxacum sp.*<br>*Medicago sativa* (alfalfa)<br>*Onobrichis sativa* (sainfoin)	overwintering hosts for the nymphs; adults aggregate mainly on *Medicago sativa* but also found on diverse crops (potato, sugar beet, sainfoin, red clover, bean and rarely sunflower and squash)	Güclü & Ozbek 1988
Israel	*Tamarix* sp.<br>*Gossypium* sp. (cotton)<br>*Solanum tuberosum* (potato)<br>*Zea mays* (corn)	adults	Hoch & Remane 1985
	Amaranthus retroflexus<br>*Convolvulus arvensis*<br>*Myrtus communis*<br>*Olea europaea,*	adults;<br>nymphs were found on the *V. agnus-castus* roots	Sharon *et al.* 2005

	Vitex agnus-castus<br>*Vitis vinifera* (grapevine)		
Greece	*Acer campestre*<br>*Arbutus* sp.<br>*Arundo* sp.<br>*Artemisia* sp.<br>*Carpinus betulus*<br>*Cistus* sp.<br>*Convolvulus* sp.<br>*Crataegus* sp.<br>*Cynodon* sp.<br>*Erica arborea*<br>*Euphorbia dendroides*<br>*Galium* sp.<br>*Hipparhenia hirta*<br>*Imperata cylindrica*<br>*Juncus* sp.<br>*Ligustrum vulgare*<br>*Myrtus* sp.<br>*Oreganum* sp.<br>*Quercus coccifera*<br>*Quercus cerris*<br>*Quercus ilex*<br>*Quercus pubescens*<br>*Paliurus* sp.<br>*Phragmites* sp.<br>*Platanus* sp.<br>*Populus* sp.<br>*Prunus spinosa*<br>*Pteridium* sp.<br>*Rubus* sp.<br>*Salix* sp.<br>*Tamarix* sp.<br>*Ulmus* sp.<br>*Vitex* sp.<br>*Capsicum anuum* (paprika)<br>*Gossypium sp.* (cotton)<br>*Sesamum indicum* (sesame)	adults;<br>the most common host plant is *Vitex* sp. in coastline habitats	Hoch & Remane 1985

	Solanum tuberosum (potato)<br>*Zea mays* (corn)		
Cyprus and Greek islands (Crete, Lesbos, Ikaria, Rhodes)	*Arbutus* sp.<br>*Arundo* sp.<br>*Erica arborea*<br>*Euphorbia dendroides*<br>*Imperata cylindrica*<br>*Juncus* sp.<br>*Phragmites* sp.<br>*Platanus* sp.<br>*Pteridium* sp.<br>*Tamarix* sp<br>*Quercus pubescens*<br>*Vitex* sp.	adults;<br>the most common host plant is *Vitex* sp. in coastline habitats	Hoch & Remane 1985
Romania	*Solanum tuberosum* (potato)	adults	Imo *et al.* 2013
Macedonia	*Convolvulus arvensis*<br>*Urtica dioica*	adults collected using sweep net and mouth aspirator	Atanasova *et al.* 2015
Montenegro	*Convolvulus arvensis*<br>*Urtica dioica*<br>*Vitex agnus-castus*	adults collected using sweep net and mouth aspirator	Kosovac *et al.* 2016
Serbia	*Convolvulus arvensis*		Aleksić *et al.* 1967
	various vineyard weeds	first data of *Crepis foetida* as a member of weed community where adults were non selectively captured by sweeping net	Cvrković Tatjana, PhD thesis, 2010
	Convolvulus arvensis<br>*Urtica dioica*<br>*Crepis foetida*	adults collected using sweep net and mouth aspirator	Kosovac *et al.* 2013
Bosnia and Herzegovina	*Convolvulus arvensis*<br>*Vitex agnus-castus*	adults collected using sweep net and mouth aspirator	Đurić *et al.* 2017
Croatia	*Vitex agnus-castus*	very often found on *V. agnus-castus*	Horváth 1891
Slovenia	*Convolvulus arvensis*<br>*Urtica dioica*	adults collected using yellow sticky traps or by sweep net sampling	Mehle *et al.* 2011

	Convolvulus arvensis<br>*Urtica dioica*	adults collected on a single plant species with an exhauster or with sweep nets in monoculture plant stands	Imo *et al.* 2013
Hungary	*Urtica dioica*	adults collected with D-Vac aspirators	Palermo *et al.* 2004
Austria	*Urtica dioica*<br>*Convolvulus arvensis*	association with *U. dioica* is dominant	Aryan *et al.* 2014
Switzerland	*Urtica dioica*<br>*Convolvulus arvensis*	adults collected with mouth aspirators mainly on *U. dioica*; nymphs present on the root in the field and confirmed as laboratory plant for nymphs development	Kessler *et al.* 2011
	Calystegia sepium<br>*Lepidium draba*<br>*Cardaria draba*<br>*Clematis vitalba*	adults were captured on yellow sticky traps positioned above the specific plant	
	Polygonum aviculare<br>*Calystegia sepium*<br>*Taraxatum officinale*<br>*Plantago lanceolata*<br>*Calystegia sepium*	adults were taken with a D-vac vacuum insect collector	
Germany	*Convolvulus arvensis*<br>*Festuca* sp.<br>*Allium* sp.	on limestone	Hoch & Remane 1985
	Convolvulus arvensis<br>*Solanum nigrum*<br>*Vitis vinifera* (grapevine)		Maixner 1994
	Ranunculus bulbosus		Weber & Maixner 1998
	Convolvulus arvensis<br>*Urtica dioica*<br>*Calystegia sepium*		Langer & Maixner 2004
France	*Calystegia sepium*<br>*Cardaria draba*<br>*Convolvulus arvensis*	adults collected by sweeping net	Sforza *et al.* 1999

	Galium verum<br>*Lavandula angustifolia*<br>*Linaria striata*<br>*Plantago cynops*<br>*Satureia montana*		
	Convolvulus arvensis<br>*Urtica dioica*	adults collected with D-Vac aspirators	Bressan *et al.* 2008
	Salvia sclarea	adults collected with D-Vac aspirators and yellow sticky traps; nymphs collected on uprooted individual plants	Chuche *et al.* 2018
	Convolvulus arvensis<br>*Urtica dioica*	adults collected with D-Vac aspirators and yellow sticky traps	
	Lavandula angustifolia (lavander)<br>*Lavandula x intermedia* (lavandin)	adults were captured on yellow sticky traps in fields planted with diverse lavender and lavandin cultivars	Yvin *et al.* 2017
	Salvia sclarea	nymphs counted on uprooted plants; adults collected with emergence traps	Hossard *et al.* 2018
	Convolvulus arvensis<br>*Lavandula hybrida*	adults collected with emergence traps	
Spain	*Trifolium pratense*		Hoch & Remane 1985
	Convolvulus arvensis		Sabate *et al.* 2014
Italy	*Urtica dioica*<br>*Convolvulus arvensis*	nymphs found on *U. dioica* roots	Lessio *et al.* 2007
Italy (Sicily)	*Dactylis* sp.<br>*Juncus* sp.<br>fam. *Lamiaceae*<br>*Ulmus* sp.<br>*Urtica* sp.		Hoch & Remane 1985
Morocco	*Clematis* sp.<br>*Pistacia* sp.<br>*Tamarix* sp<br>*Quercus coccifera*		Hoch & Remane 1985

References

Atanasova B, Jakovljević M, Spasov D, Jović J, Mitrović M, Toševski I, Cvrković T (2015) The molecular epidemiology of bois noir grapevine yellows caused by '*Candidatus* Phytoplasma solani'in the Republic of Macedonia. *European Journal of Plant Pathology*, **142**, 759-770.

Aleksić Ž, Šutić D, Aleksić D (1967) Transmission intensity of stolbur virus by means of *Hyalesthes obsoletus* Sign. on some host plants. *Plant Protection*, **93–95**, 67–73.

Aryan A, Brader G, Mörtel J, Pastar M, Riedle-Bauer M (2014) An abundant '*Candidatus* Phytoplasma solani' Stolbur tuf b phytoplasma strain is associated with grapevine, stinging nettle and *Hyalesthes obsoletus*. *European Journal of Plant Pathology*, **140**, 213-227.

Bressan A, Sémétey O, Nusillard B, Clair D, Boudon-Padieu E (2008) Insect vectors (Hemiptera: Cixiidae) and pathogens associated with the disease syndrome "basses richesses" of sugar beet in France. *Plant Disease*, **92**(1), 113-119.

Chuche J, Danet JL, Rivoal JB, Arricau-Bouvery N, Thiéry D (2018) Minor cultures as hosts for vectors of extensive crop diseases: Does *Salvia sclarea* act as a pathogen and vector reservoir for lavender decline? *Journal of Pest Science*, 91(1), 145-155.

Cvrković T (2010) Diverzitet faune cikada u vinogradima Srbije i njihova uloga u prenošenju *Bois Noir* fitoplazme. PhD thesis, Faculty of Agriculture, University of Belgrade, 1-101.

Đurić Z, Hrnčić S, Delić D (2017) Morphological and molecular identification of *Hyalesthes obsoletus* Signoret (Auchenorrhyncha: Cixiidae) in Herzegovina vineyard. *Mitteilungen Klosterneuburg* **67**, 177-181

Güclü S, Ozbek H (1988) Some studies on the biology of *Hyalestes obsoletus* Signoret (Homoptera: Cixidae) in the conditions of Erzurum. *Turkiye-Entomoloji-Dergisi*, **12**, 103-111.

Hoch H, Remane R (1985) Evolution und Speziation der Zikaden-Gattung Hyalesthes Signoret, 1865 (Homoptera Auchenorrhyncha Fulgoroidea Cixiidae). *Marburger Entomologische Publikationen*, **2**, 1-427.

Horváth G (1891) Hemipterološki izlet u Primorje i Plitvička jezera. *Glasnik Hrvatskoga Naravoslovnoga Drustva (Societas historico-naturalis Croatica)*, VI (1-5), 29-49.

Hossard L, Guimier S, Vinatier F, Barbier JM, Delmotte S, Fontaine M, Rivoal JB (2018) Source of *Hyalesthes obsoletus* Signoret planthopper (Hemiptera: Cixiidae) in southern France and potential effects of landscape. *Bulletin of Entomological Research*, **108**, 213-222.

Imo M, Maixner M, Johannesen J (2013) Sympatric diversification vs. immigration: deciphering host-plant specialization in a polyphagous insect, the stolbur phytoplasma vector *Hyalesthes obsoletus* (Cixiidae). *Molecular Ecology*, **22**, 2188-2203.

Kessler S, Schaerer S, Delabays N, Turlings TC, Trivellone V, Kehrli P (2011) Host plant preferences of *Hyalesthes obsoletus*, the vector of the grapevine yellows disease 'bois noir', in Switzerland. *Entomologia Experimentalis et Applicata*, **139**, 60-67.

Kosovac A, Johannesen J, Krstić O, Mitrović M, Cvrković T, Maixner M, Toševski I, Jović J (2013) Microsatellite and mtDNA evidence of genetic differentiation in *Hyalesthes obsoletus* populations associated with a new major host, stinking hawk's-beard (*Crepis foetida*), in southeast Europe. *Proceedings of the 3rd European Bois Noir Workshop*, Barcelona, 18-19.

Kosovac A, Radonjić S, Hrnčić S, Krstić O, Toševski I, Jović J (2016) Molecular tracing of the transmission routes of bois noir in Mediterranean vineyards of Montenegro and experimental evidence for the epidemiological role of *Vitex agnus-castus* (Lamiaceae) and associated *Hyalesthes obsoletus* (Cixiidae*). Plant Pathology,* **65**, 285-298.

Langer M, Maixner M (2004) Molecular characterisation of grapevine yellows associated phytoplasmas of the stolbur group based on RFLP analysis of non-ribosomal DNA. *Vitis*, **43**, 191–199.

Lessio F, Tedeschi R, Alma A (2007) Population dynamics, host plants and infection rate with Stolbur phytoplasma of *Hyalesthes obsoletus* Signoret in north-western Italy. *Journal of Plant Pathology*, **89**, 97-102.

Maixner M (1994) Research note: Transmission of German grapevine yellows (Vergilbungskrankheit) by the planthopper *Hyalesthes obsoletus* (Auchenorrhyncha: Cixiidae). *Vitis*, **33**, 103-104.

Palermo S, Elekes M, Botti S, Ember I, Alma A, Orosz A, Bertaccini A, Kölber M (2004). Presence of stolbur phytoplasma in Cixiidae in Hungarian vineyards. Vitis, **43**(4), 201-203.

Sabaté J, Laviña A, Batlle A (2014) Incidence of Bois Noir phytoplasma in different viticulture regions of Spain and Stolbur isolates distribution in plants and vectors. *European Journal of Plant Pathology*, **139**, 185-193.

Sforza R, Bourgoin T, Wilson SW, Boudon-Padieu E (1999) Field observations, laboratory rearing and descriptions of immatures of the planthopper *Hyalesthes obsoletus* (Hemiptera: Cixiidae). *European Journal of Entomology*, **96**, 409–418.

Sharon R, Soroker V, Wesley SD, Zahavi T, Harari A, Weintraub PG (2005) *Vitex agnus-castus* is a preferred host plant for *Hyalesthes obsoletus*. *Journal of Chemical Ecology*, **31**, 1051-1063.

Suhov KS, Vovk AM (1949) Stolbur of solanaceous plants. USSR Academy of Sciences Publishers. 7-11.

Weber A, Maixner M (1998) Survey of populations of the planthopper *Hyalesthes obsoletus* Sign.(Auchenorrhyncha, Cixiidae) for infection with the phytoplasma causing grapevine yellows in Germany. *Journal of Applied Entomology*, **122**, 375-381.

Yvin C, Chaisse E, Fontaine M, Rivoal JB, Verdin E, Nicolè F, Jourdan P, Savarit P, Gallois P (2017) Dépérissement de la lavande et du lavandin: mise en oeuvre d'un programme de recherches appliquées afin d'apporter des solutions de lutte aux producteurs. *Innovations Agronomiques*, **55**, 107-120.
