# Supplementary material for: Widespread plant specialization in the polyphagous planthopper Hyalesthes obsoletus (Cixiidae), a major vector of stolbur phytoplasma: Evidence of cryptic speciation
Source: PLoS One. 2018 May 8;13(5):e0196969. doi: 10.1371/journal.pone.0196969 (PMC5940214; doi:10.1371/journal.pone.0196969)
Supplement: S2 Table — (PDF) [file pone.0196969.s004.pdf]

S2 Table Primers used for amplification of mitochondrial *COI-tRNA(Leu)-COII* and *16S-tRNA(Leu)-ND1* gene regions in *Hyalesthes obsoletus* and archival paratype specimen of *H. thracicus*.

Gene region	Primer name	Primer sequence (5'→3')	Short amplicon length (with primers)	Main amplicon length (with primers)
COI-tRNA(Leu)-COII	S2792	ATACCTCGACGTTATTCAGA	291 bp	S2792/A3661 898 bp
	Hy-TL-R1*	GRTTTWCTTTMTAGAYGAATTTTAAG		
	Hy-TL-F1*	TGGCAGAAAAGTGCTRTGAA	383 bp	
	Hy-COII-R1*	TCATATCTTCARTATCATTGATG		
	Hy-COII-F1*	CCATCAATTTCDATYAAAACHATAGG	320 bp	
	A3661	CCACAAATTTCTGAACATTGACCA		
16S-tRNA(Leu)-ND1	LR-N-12945	CGACCTCGATGTTGAATTAA	347 bp	LR-N-12945/ N1-J-12261 671 bp
	Hy-ND1-R1*	TTWCGAAAYTGAATATAWCC		
	Hy-ND1-F1*	GTTGCTTAYTTTATTCTTCTTGA	380 bp	
	N1-J-12261	TCGTAAGAAATTATTTGAGC		

*Degenerate primers designed in this study for amplification of short fragments in the archival specimen of *H. thracicus*. Design based on sequence alignment comparison among *Hyalesthes philesakis*, *H. luteipes*, *H. ponticorum*, *H. aylanus*, *Reptalus panzeri*, *R. melanochaetus*, *R. cuspidatus* and *Setapius apiculatus*.
