# Supplementary material for: Widespread plant specialization in the polyphagous planthopper Hyalesthes obsoletus (Cixiidae), a major vector of stolbur phytoplasma: Evidence of cryptic speciation
Source: PLoS One. 2018 May 8;13(5):e0196969. doi: 10.1371/journal.pone.0196969 (PMC5940214; doi:10.1371/journal.pone.0196969)
Supplement: S3 Table — (PDF) [file pone.0196969.s005.pdf]

S3 Table List of *Hyalesthes obsoletus* newly identified haplotypes of the two mtDNA gene regions with corresponding GenBank accession numbers and data on host-plant associations.

Gene region	Haplotype†	NCBI Accesion number	Associated host-plant
COI-tRNA(Leu)-COII	S	KY368699	*Convolvulus arvensis*
	X	KY368700	*Convolvulus arvensis*
	π (pi)	KY368701	*Convolvulus arvensis*
	ψ (psi)	KY368702	*Convolvulus arvensis*
	α (alpha)	KY368703	*Urtica dioica*
	β (beta)	KY368704	*Urtica dioica*
	ξ (xi)	KY368705	*Urtica dioica*
	R	KY368706	*Urtica dioica*
	ρ (rho)	KY368707	*Urtica dioica*
	ω (omega)	KY368708	*Urtica dioica*
	W	KY368709	*U. dioica, C. arvensis*
	J	KY368710	*Crepis foetida*
	M	KY368711	*Crepis foetida*
	U	KY368712	*Crepis foetida*
	V	KY368713	*Crepis foetida*
	λ (lambda)	KY368714	*Crepis foetida*
	μ (mu, micro)	KY368715	*Crepis foetida*
	Y	KY368716	*Vitex agnus-castus*
	Z	KY368717	*Vitex agnus-castus*
	γ (gamma)	KY368718	*Vitex agnus-castus*
	η (eta)	KY368719	*Vitex agnus-castus*
	σ (sigma)	KY368720	*Vitex agnus-castus*
	θ (theta)	KY368721	*Vitex agnus-castus*
	P	KY368722	crop plant
	ϕ (phi)	KY368723	crop plant
	Q	KY368724	crop plant
16S-tRNA(Leu)-ND1	K	KY368692	*Convolvulus arvensis*
	L	KY368693	*Convolvulus arvensis*
	Q	KY368694	*Urtica dioica*
	H	KY368695	*Crepis foetida*
	M	KY368696	*Vitex agnus-castus*
	N	KY368697	*Vitex agnus-castus*
	O	KY368698	*Vitex agnus-castus*

† Letter names for Greek alphabet are given in parentheses
