# Supplementary material for: Widespread plant specialization in the polyphagous planthopper Hyalesthes obsoletus (Cixiidae), a major vector of stolbur phytoplasma: Evidence of cryptic speciation
Source: PLoS One. 2018 May 8;13(5):e0196969. doi: 10.1371/journal.pone.0196969 (PMC5940214; doi:10.1371/journal.pone.0196969)
Supplement: S4 Table — (PDF) [file pone.0196969.s006.pdf]

S4 Table Neutrality tests for the *Hyalesthes obsoletus* host-plant grouped populations and the overall dataset based on mtDNA *COI-tRNA(Leu)-COII* and *16S-tRNA(Leu)-ND1* data.

Dataset (N)	n	Neutrality tests	
		Tajima's *D*	Fu's F_S
Crepis foetida (83)	6	-1.04433 (p = 0.16800)	-2.56721 (p = 0.07400)
Vitex agnus-castus (56)	8	1.95886 (p = 0.97300)	2.65819 (p = 0.84600)
Convolvulus arvensis (57)	8	-0.43667 (p = 0.39500)	-0.54393 (p = 0.44400)
Urtica dioica (120)	9	-2.18859 (p = 0.00100)***	-8.59152 (p = 0.00000)***
Convolvulus-Urtica (177)	15	-1.87421 (p = 0.00500)**	-9.59790 (p = 0.00100)***
Total (316)	29	-0.31990 (p = 0.38200)	-3.16897 (p = 0.30700)

n, number of haplotypes.

*p < 0.05; **p < 0.01; ***p < 0.001.
