# Supplementary material for: Widespread plant specialization in the polyphagous planthopper Hyalesthes obsoletus (Cixiidae), a major vector of stolbur phytoplasma: Evidence of cryptic speciation
Source: PLoS One. 2018 May 8;13(5):e0196969. doi: 10.1371/journal.pone.0196969 (PMC5940214; doi:10.1371/journal.pone.0196969)
Supplement: S5 Table — (PDF) [file pone.0196969.s007.pdf]

S5 Table Average evolutionary distances calculated for the *COI-COII* gene region (711 bp) for 37 *H. obsoletus* haplotypes (excluding the Q, ϕ and P, and the Israeli G and H derived haplotypes), corrected by applying the HKY+I substitution model. Range values are placed in brackets. N-number of haplotypes within each group; *d*1-average divergence among all sequence pairs; *d*2-average divergence among sequence pairs within host-plant associated groups; *D*-average divergence among sequence pairs between groups.

Hyalesthes obsoletus host-plant associated haplotype group	N	*d1*	*d2*	*D*			
				1	2	3	4
1. *ex Convolvulus*	12	0.010 (0.000-0.027)	0.004 (0.001-0.009)	-			
2. *ex Urtica*	17		0.004 (0.001-0.007)	0.004 (0.000-0.009)	-		
3. *ex Vitex*	6		0.005 (0.001-0.011)	0.018 (0.010-0.025)	0.016 (0.009-0.021)	-	
4. *ex Crepis*	6		0.003 (0.001-0.004)	0.020 (0.014-0.027)	0.018 (0.012-0.025)	0.012 (0.007-0.017)	-
Hyalesthes thracicus	-	-	-	0.038 (0.033-0.043)	0.037 (0.031-0.043)	0.029 (0.025-0.032)	0.029 (0.025-0.032)
