# Supplementary material for: Widespread plant specialization in the polyphagous planthopper Hyalesthes obsoletus (Cixiidae), a major vector of stolbur phytoplasma: Evidence of cryptic speciation
Source: PLoS One. 2018 May 8;13(5):e0196969. doi: 10.1371/journal.pone.0196969 (PMC5940214; doi:10.1371/journal.pone.0196969)
Supplement: S6 Table — (PDF) [file pone.0196969.s008.pdf]

S6 Table AMOVA *F*-statistical comparison of genetic divergence within and between *Hyalesthes obsoletus* populations grouped according to host-plant associations or geography. The results present the percent of total among-population divergence that can be attributed to groups (F_{CT}) or to the within-group variance (F_{SC}).

Analysis	Overall genetic variance	Variance within group	Variance between group (host-plant or geography)	% of the total variance attributed to host-plant or geography
	F_{ST}	F_{SC}	F_{CT}	F_{CT}/F_{ST} x 100%
All 50 *H. obsoletus* populations genotyped in this study and single population associated with *V. agnus-castus* from Israel, grouped according to host-plant	0.180***	0.047***	0.139***	77%
Montenegrin and Greek *H. obsoletus* populations from *V. agnus-castus*, grouped according to regional affiliation	0.097***	0.041***	0.059*	60%
Montenegrin and Greek *H. obsoletus* populations from *V. agnus-castus* and single population associated with *V. agnus-castus* from Israel, grouped according to regional affiliation (2 clusters) as classified by STRUCTURE	0.108***	0.064***	0.046**	42%

*p < 0.05; **p < 0.01; ***p < 0.001.
